# Mitochondrial, exosomal miR137-COX6A2 and gamma synchrony as biomarkers of parvalbumin interneurons, psychopathology, and neurocognition in schizophrenia

**DOI:** 10.1038/s41380-021-01313-9

**Published:** 2021-10-22

**Authors:** Ines Khadimallah, Raoul Jenni, Jan-Harry Cabungcal, Martine Cleusix, Margot Fournier, Elidie Beard, Paul Klauser, Jean-François Knebel, Micah M. Murray, Chrysa Retsa, Milena Siciliano, Kevin M. Spencer, Pascal Steullet, Michel Cuenod, Philippe Conus, Kim Q. Do

**Affiliations:** 1 Center for Psychiatric Neuroscience, Department of Psychiatry, Lausanne University Hospital (CHUV), Lausanne, Switzerland; 2 Service of General Psychiatry, Department of Psychiatry, University Hospital Center and University of Lausanne, Prilly Lausanne, Switzerland; 3 The LINE (Laboratory for Investigative Neurophysiology), Radiodiagnostic Service, University Hospital Center and University of Lausanne, 1011 Lausanne, Switzerland; 4 Sensory, Perceptual and Cognitive Neuroscience Section, Center for Biomedical Imaging (CIBM), University Hospital Center and University of Lausanne, 1011 Lausanne, Switzerland; 5 Ophthalmology Department, Fondation Asile des Aveugles and University of Lausanne, Lausanne, Switzerland; 6 Neural Dynamics Laboratory, Research Service, Veterans Affairs Boston Healthcare System, Boston, MA USA; 7 Department of Psychiatry, Harvard Medical School, Boston, MA USA

**Keywords:** Diagnostic markers, Molecular biology, Diagnostic markers

## Abstract

Early detection and intervention in schizophrenia requires mechanism-based biomarkers that capture neural circuitry dysfunction, allowing better patient stratification, monitoring of disease progression and treatment. In prefrontal cortex and blood of redox dysregulated mice (*Gclm*-KO ± GBR), oxidative stress induces miR-137 upregulation, leading to decreased COX6A2 and mitophagy markers (NIX, Fundc1, and LC3B) and to accumulation of damaged mitochondria, further exacerbating oxidative stress and parvalbumin interneurons (PVI) impairment. MitoQ, a mitochondria-targeted antioxidant, rescued all these processes. Translating to early psychosis patients (EPP), blood exosomal miR-137 increases and COX6A2 decreases, combined with mitophagy markers alterations, suggest that observations made centrally and peripherally in animal model were reflected in patients’ blood. Higher exosomal miR-137 and lower COX6A2 levels were associated with a reduction of ASSR gamma oscillations in EEG. As ASSR requires proper PVI-related networks, alterations in miR-137/COX6A2 plasma exosome levels may represent a proxy marker of PVI cortical microcircuit impairment. EPP can be stratified in two subgroups: (a) a patients’ group with mitochondrial dysfunction “Psy-D”, having high miR-137 and low COX6A2 levels in exosomes, and (b) a “Psy-ND” subgroup with no/low mitochondrial impairment, including patients having miR-137 and COX6A2 levels in the range of controls. Psy-D patients exhibited more impaired ASSR responses in association with worse psychopathological status, neurocognitive performance, and global and social functioning, suggesting that impairment of PVI mitochondria leads to more severe disease profiles. This stratification would allow, with high selectivity and specificity, the selection of patients for treatments targeting brain mitochondria dysregulation and capture the clinical and functional efficacy of future clinical trials.

## Introduction

Among the most perdurable observations in schizophrenia (SZ) is a functional impairment of cortical parvalbumin interneurons (PVIs) [1]. This subset of GABAergic cells controls the precise synchronous activity of pyramidal neuron ensembles by virtue of their high firing rates within the cortical network and thereby modulates gamma-frequency rhythmicity and its cognitive correlates [1]. Defects in PVIs are reported to underlie the disruption of neural synchrony and subsequent fragmentation of cognitive-behavioral processes typically observed in SZ [2]. To sustain the large energy demand required to drive the high firing rate and rhythmicity of PVIs, these cells possess high mitochondrial content [3], which renders them more susceptible to oxidative stress (OxS) [4]. Mounting evidence suggests the presence of mitochondrial dysfunction in SZ, as indicated by the loss of electron transport chain activity and a shift toward anaerobic activity [5]. However, the precise molecular and cellular mechanisms that would allow a translation from animal models to patients are still missing. Moreover, mechanism-based blood biomarkers mediating specific behavioral and cognitive alterations are needed for patient stratification in terms of treatments and prevention.

Recent research points to OxS as one “central hub” in SZ pathophysiology, with converging evidence from environmental and genetic studies linking this process to PVI impairment [6]: indeed, in series of animal models carrying either genetic and/or environmental risks, we showed that PVI deficits were all accompanied by oxidative stress in prefrontal cortex. OxS may result from dysregulation of systems typically affected in SZ, including glutamatergic, dopaminergic, immune, and antioxidant signaling. As convergent end-point, redox dysregulation has successfully been targeted to protect PVIs with antioxidants/redox regulators across several animal models [6–8]. More importantly, the antioxidant and glutathione precursor NAC gave promising results in the improvement of cognition in early psychosis patients [8–12].

Notably, disturbances in endogenous antioxidants levels, particularly decreased glutathione (GSH) levels were reported in blood, CSF, and prefrontal cortex of SZ patients [8, 13–15]. Furthermore, variants of the genes for both modulatory (GCLM) and catalytic (GCLC) subunits of the key GSH-synthesizing enzyme glutamate-cysteine-ligase (GCL) were associated with SZ [16–19]. Accordingly, cultured skin fibroblasts of individuals expressing high-risk GAG-trinucleotide repeat (TNR) polymorphisms in the GCLC gene have decreased the GCLC protein expression, GCL activity, and GSH content. In addition, SZ associated polymorphisms and copy number variations of genes related directly to antioxidant system, lead to increased vulnerability to OxS [20–23]. However, these association studies based on relatively small number of subjects would deserve to be validated in larger cohorts.

To explore biological mechanisms underlying phenotypes such as cognitive deficits, transgenic *Gclm*-KO + GBR mice were developed as a model of a genetic risk (GSH displaying 70% GSH deficit) combined with environmental risk (insults, stress, trauma) at various timing during the development [12, 24, 25]. Indeed, beyond exhibiting cortical deficiencies in PVIs [26–29] and alterations of beta/gamma oscillations, *Gclm*-KO mice display elevated levels of the oxidative DNA damage marker 8-oxo-7,8-dihydro-2′-deoxyguanosine (8-oxo-dG) in the extranuclear compartment. This is exacerbated by additional insults in *Gclm*-KO mice challenged with GBR12909 (GBR), a specific dopamine reuptake inhibitor used to mimic the increased dopamine signaling known to generate reactive oxygen species (ROS) through its catabolism, as observed in SZ. The 8-oxo-dG extranuclear localization is suggestive of mitochondrial DNA oxidation [25], potentially related to impaired mitophagy inducing an accumulation of defective mitochondria [30]. Indeed, mitophagy, critical for basal mitochondrial turnover, is also known as a “cleaning system” and is induced as a stress-response mechanism to eliminate damaged mitochondria, thus avoiding cellular apoptosis [31]. Mitophagy processing is mediated by the autophagy receptors BCL2 Interacting Protein 3 Like (Nix/BNIP3L), BCL2 Interacting Protein3 (BNIP3), and FUN14 Domain Containing 1 (FUNDC1), which are responsible for delivery to autophagosomes by binding to Microtubule-associated protein1A/1B-light chain3 (LC3) [32]. In addition to its important role in the regulation of synaptic function, including synaptogenesis, spine development and morphology [33, 34]^,^, miR-137, a 23-nt noncoding microRNA located on chromosome 1, has been reported to be critically involved in mitophagy [35]. Mitophagy is under the post-transcriptional control of miR-137. Indeed, miR-137 inhibits mitophagy by controlling Fundc1 and Nix expression [35]. MiR-137 expression is enriched in the mouse and human brain, with high expression in the cortex and hippocampus and low expression in the cerebellum and brain stem [34].

Here, we investigated the impact of redox dysregulation on mitophagy deficits in PVIs using the *Gclm*-KO model. The role of OxS was examined using a quantitative assessment of markers for OxS, PVIs, and mitophagy in the anterior cingulate cortex (ACC), a prefrontal area known to be affected and to display redox dysregulation in SZ [36]. We then investigated the effects of redox dysregulation on miR-137 expression given its involvement in mitophagy regulation [35] and robust evidence of genetic association with SZ [37]. We also investigated cytochrome c oxidase subunit VIa polypeptide2 (COX6A2), a subunit of cytochrome c oxidase complex IV (COX-IV), the terminal enzyme in the mitochondrial respiratory chain [38]. Indeed, altered function of COX, mainly related to dysregulation of mitochondrial gene expression, underlies mitochondrial dysfunction in brain tissue [39].

We next explored whether mitochondria-targeted antioxidant treatment with mitoquinone mesylate (MitoQ) can restore PVI integrity and mitophagy deficits. Finally, by adopting a reverse translational approach, we sought to validate our preclinical observations in a cohort of patients in the early phase of psychosis (EPP) to determine the potential mechanistic pathway through which disturbed mitophagy may affect PVI-mediated gamma oscillations as assessed by the auditory steady-state response (ASSR) in EEG, which are critically involved in cognitive performance [2, 40].

## Materials, subjects, and methods

### Preclinical, mice study

The tissue preparation, immunohistochemistry, imaging and image processing, microRNA in situ hybridization of transgenic mice lacking the glutamate-cysteine ligase modifier subunit (Gclm-KO; B6.129-Gclmtm1Tdal crossbred with C57BL/6J mice over more than 10 generations) [26] as well as the electron microscopy study and the quantification of plasmatic miR-137 were described in Supplementary materials. All animal procedures were conducted in accordance with the guidelines outlined in the Guide for the Care and Use of Laboratory Animals and were approved by the Local Veterinary Office.

#### Reversal study with MitoQ

The selective mitochondrial antioxidant MitoQ has shown promise in preclinical models of disease related to mitochondrial dysfunction, such as the EAE model or multiple sclerosis, a condition in which some people may develop psychotic symptoms [41]. Half of the animals from each litter (4 females and 4 males/Group) received a single subcutaneous (s.c.) injection daily with GBR12909 (GBR) from P10 to P20. As a control, the other half of the animals received a single injection daily with phosphate buffered saline. Thereafter, from P21 to P40, half of the animals were treated with a MitoQ solution (500 nM, dissolved in water), and the other half were treated with water. The MitoQ solution was freshly prepared every other day and delivered through water bottles that were light-protected to prevent rapid oxidation. The mice were then sacrificed at P40.

#### Local reversal study with miR-137 inhibitor

##### miRNA solution

Two solutions of miRNA Inhibitor (miRCURY LNA miRNA custom Inhibitor in vivo large scale, Qiagen) were used in this study, one with our specific sentence of MIR-137 (CGTATTCTTAAGCAAT) and one with a scrambled sentence (as a sham). The final concentration of our solution was at 0.24 nmol/500 nl per injection.

##### miRNA injection

Mice 6–8 week old were anesthetized using Ketamine-Xylazine (83 and 3.5 mg/kg, respectively) and placed on a heating blanket to maintain the body temperature at 37 °C. The animal was head fixed on a stereotaxic apparatus (Kopf model 940, Tujunga, CA) to perform the surgery.

The bone was exposed at the desired position through a small skin incision and small craniotomies (<0.5-mm diameter) were performed above the site of injection at (anteroposterior (AP), mediolateral (ML), depth from cortical surface (DV), in stereotaxic coordinates from Bregma): 0.7; ±0.3; −1.2 to target the ACC. Each solution of miRNA were injected with a thin glass pipette (5-000-1001-X, Drummond Scientific, Broomall, PA) pulled on a vertical puller (Narishige PP-830, Tokyo, Japan).

At the end of the surgery, mice received a dose of analgesic (Buprenorphine, s.c. 0.1 mg/kg body weight). After 4 days, mice were injected i.p. with a lethal dose of penthobarbital and intracardial injection of about 50 ml of paraformaldehyde 4% was done to collect the organs. The brains were collected in PBS 0.1 M 30% sucrose overnight at 4 °C and sliced with a microtome (Microm HM440E, section thickness: 60 μm). The slices were disposed in 12-well plates filled with 0.1 M PB for immunohistochemistry.

### Human clinical study

This study was carried out in accordance with the Declaration of Helsinki and was approved by the local Ethics Committee (*Commission cantonale d’éthique de la recherche sur l’être humain (CER-VD)*). Written documentation of informed consent for this study was obtained from each participant.

#### Subjects recruitment

The study population included early psychosis patients (EPP; *n* = 138) and healthy controls (*n* = 134), matched for gender and age (Table S1). The patients were recruited from the Treatment and Early Intervention in Psychosis Program [42], which is a specialized 3-year program for the treatment of early psychosis patients. The inclusion/ exclusion criteria as well as the clinical, functional, and neurocognitive assessments were detailed in Supplementary materials.

#### Blood markers analysis

***Blood collection and redox marker processing*** were previously described [43] and detailed in Supplementary materials.

***Quantification of circulating miR-137 (ExomiRs)***. *Exosome purification*: To isolate exosomes from human plasma, polycarbonate tubes (Beckman Coulter) containing 200 µl of plasma were centrifuged twice at 100,000 *g* at 4 °C for 70 min using ultracentrifugation (Sorvall ultra Pro 80). miRNA extraction, reverse transcription, and qPCR amplification were detailed in Supplementary materials.

#### Exosome extraction and staining

Exosome isolation was performed on plasma samples using an ExoQuick Kit according to the provided instructions. The total isolated exosomes were then used for immunostaining purposes performed on a panel of exosomal (CD9, HSP70, L1CAM; for more details, see Table S2) and nonexosomal markers (COX6A2 and parvalbumin).

***Quantification of plasma levels of mitophagy markers using Western blot*** (see Supplementary materials).

***Quantitative determination of the exosome levels of COX6A2*** (see Supplementary materials).

### Electroencephalography study

A subsample of the above-described cohort, 33 patients and 33 matched healthy controls, participated in an EEG study (Table S1). The procedures of the EEG stimulation, acquisition, preprocessing, time-frequency decomposition, and statistical analyses were detailed in the Supplementary materials.

**Quantification and statistical analysis** (see Supplementary materials).

## Results

### Oxidative stress elicits mitophagy in the ACC of *Gclm-KO* mice

**Fig. 1:**
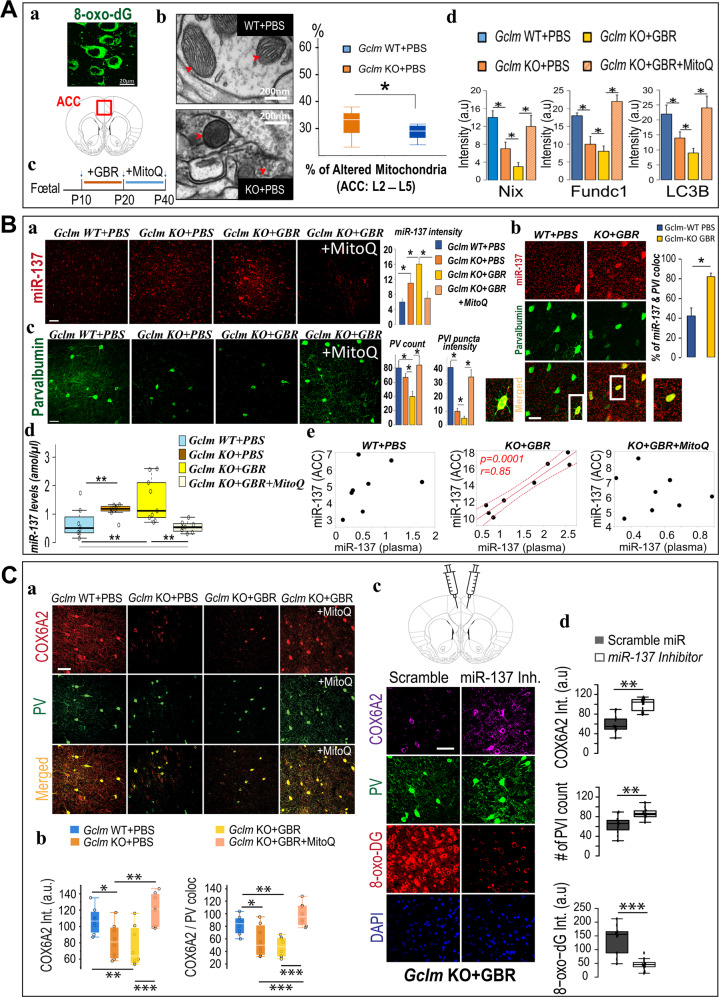
Observations in *Gclm-KO* mice. **A** Redox dysregulation affects mitochondrial morphology and reduces the expression of mitophagy markers in ACC. (**a**) 8-oxo-dG labeling of mitochondrial DNA in the ACC. Scale bar 20 μm. (**b**) Electron micrographs show mitochondrial morphology and mitophagosomes (red arrowheads) in *Gclm*-KO and *Gclm*-WT mice. Scale bar 200 nm. (**c**) Time course of pharmacological challenge with GBR (5 mg/kg, s.c. inj.) followed by MitoQ treatment (500 µmol/L, drinking water). (**d**, **e**) Mitophagy markers (Nix, Fundc1, and LC3B) labeling in ACC across four different groups. Scale bar 50μm. *p* < 0.05*, see representative micrograph in SI (Fig. S1A). **B** Expression of miR-137 and PV in ACC neurons. (**a**) Representative micrographs of miR-137 labeling in ACC across four different groups. Scale bar 50μm. (**b**) Micrographs showing double labeling for PV (green) and miR-137 (red) in ACC in *Gclm-KO* + *GBR* and *Gclm-WT* + *PBS* mice at P40. Scale bar 50 μm. (**c**) Representative micrographs of PV labeling in ACC across the four different groups. Scale bar 50μm. (**d**) The increased plasmatic levels of miR-137 in mice *Gclm* KO ± GBR) were rescued by mitoQ treatment (*Gclm* KO + GBR + MitoQ) back to baseline level, as observed in control animals (*Gclm* WT + PBS). (**e**) Positive correlation between miR-137 plasma and brain levels in *Gclm* KO + GBR (center panel), reversed by mitoQ treatment (*Gclm* KO + GBR + MitoQ; right panel). *p* < 0.01**, *p* < 0.05*. **C** Expression of COX6A2 in PV neurons in the ACC. (**a**) Colocalization labeling for COX6A2 (red) and PV (green) in ACC was increased in *Gclm* KO + GBR after mitoQ treatment. Scale bar 50 μm. (**b**) Decreased COX6A2 labeling intensity and COX6A2 / PV colocalization in ACC of P40 mice with or without GBR. (**c**, **d**) Local injection of miR-137 inhibitor (i-mmu-miR-137; miRCURY inhibitor Qiagen) in one hemisphere and scramble miR in the contralateral side; immunolabeling for COX6A2 (purple), PV (green), 8-oxo-dG (red), and Dapi (blue) in ACC of *Gclm* KO + GBR mice (**c**). Inhibition of miR-137 led to decreased oxidative stress marker 8-oxo-DG and increased PVI and COX6A2 staining in *Gclm*-KO + GBR as compared to sham injection (**d**). *p* < 0.001***, *p* < 0.01**, *p* < 0.05*.

Pharmacological challenge of *Gclm*-KO mice with GBR (*Gclm*-KO + GBR) induces elevated levels of the oxidative DNA damage marker 8-oxo-dG in their ACC [25, 44]. Based on its extranuclear localization (Fig. 1Aa), we surmised that mitochondrial DNA (mtDNA) was affected and probed this idea at the ultrastructural level. In the ACC of *Gclm*-KO + PBS mice (P20) (Fig. 1Ab), both qualitative and quantitative assessment of electron micrographs revealed increased numbers of altered mitochondria with a darker shading and a more globular shape, indicative of damaged mitochondria [30], suggestive of impaired mitophagy [30, 45] in particular in case of damaged mitochondria localized in neuropiles [35% in *Gclm*-KO mice versus 19% in *Gclm*-WT mice]. The principal markers of mitophagy, including NIX, Fundc1, and LC3B, were decreased in the *Gclm*-KO + GBR compared to WT mice (Fig. 1Ad and Fig. S1A). In comparison with those of WT mice (*Gclm*-WT + PBS), the levels of the mitophagy receptors NIX (BNIP3L) and Fundc1 and of the autophagosome LC3B were reduced in the ACC of *Gclm*-KO mice (*Gclm*-KO + PBS, at P40). Additional oxidative insult with GBR (P10-P20, Fig. 1Ac) further decreased NIX (Fig. S1A, Fig. S1B), Fundc1 and LC3B staining intensity (Fig. 1Ad and Fig. S1A). Taken together, these observations are consistent with an oxidative stress-induced mitophagy deficit in the ACC.

### Altered miR-137 expression in relation to PVI integrity in the ACC of *Gclm*-KO mice

The microRNA miR-137 plays a functional role in modulating synaptic function [33, 46] and in regulating the expression of the NIX and Fundc1 mitophagy receptors [35]. In *Gclm*-KO mice compared to WT animals, the combined use of in situ hybridization of miR-137 and immunohistochemistry of PVI revealed a three-fold increase expression level of miR-137. (Fig. 1Ba, Fig. S2). Although miR-137 was expressed in processes of various cell types, additional GBR oxidative challenge induced a marked increase, at somatic level, in the colocalization of miR-137 staining exclusively in PVIs, suggesting that the observed miR-137 overexpression was localized mainly in PVIs, (Fig. 1Bb). Consistent with previous results [44], PV immunoreactive cell soma counts and puncta-related process intensity were decreased in *Gclm*-KO compared to those in the WT mice (−12.5% and −75%, respectively) and were further reduced in *Gclm*-KO + GBR animals (−50% and −87.5%, respectively) (Fig. 1Bc). These findings demonstrate that in transgenic animals susceptible to redox dysregulation challenged with additional insults, miR-137 was upregulated, leading to decreased mitophagy and a subsequent accumulation of damaged mitochondria that further exacerbates OxS and PVI impairment.

### Increased plasma level of miR-137 in *Gclm-KO mice*

To test whether the observed alterations of prefrontal miR-137 can be observed in periphery, its plasmatic levels were quantified and related to central levels. In *Gclm*-KO mice (±GBR), miR-137 plasma levels were increased as compared to WT (Fig. 1Bd; see also Fig. S3 for the other groups). miR-137 plasma and ACC levels correlated positively in *Gclm*-KO + GBR mice (Fig. 1Be). This association between central and peripheral miR-137 levels was absent in WT and in KO + GBR treated with MitoQ. (Fig. 1Bd), this suggests that miR-137 plasma levels in *Gclm*-KO + GBR animals may reflect their elevated central levels.

### Brain-specific colocalization of COX6A2 and PV

To characterize mitochondrial status in ACC, we performed several staining procedures with various mitochondrial markers (listed in Table S2). Among them, the subunit COX6A2 of complex IV was selected for its specific colocalization to PVIs (Fig. 1Ca, Fig. S4). At P40, COX6A2 was decreased by 22% in *Gclm-*KO mice and by 41% in *Gclm*-KO-GBR (Fig. 1Cb). The labeling of parvalbumin and COX6A2 was colocalized in both the soma and neuropil of the ACC (Fig. 1Ca), suggesting that COX6A2 is predominantly present in fast-spiking PVIs.

### MitoQ treatment rescues oxidative stress-induced miR-137, COX6A2, mitophagy, and PVI alterations

In view of the detrimental effects of OxS on PVI integrity, miR-137 expression level, COX6A2 protein level, and mitophagy, we evaluated whether treatment with MitoQ, a selective mitochondria-targeted antioxidant [41], could rescue these deficits in young KO mice. Treatment with MitoQ (P21-P40) in *Gclm*-KO + GBR mice (Fig. 1Ac) normalized to WT values of Nix, Fundc1 and LC3B expression levels (Fig. 1Ad), miR-137 expression levels (plasma and ACC) (Fig. 1Ba, d) and COX6A2 staining intensity (Fig. 1Ca,d). Additionally, abnormalities in cell body counts and staining intensity of PVI processes in ACC of *Gclm*-KO + GBR animals were restored to WT levels (Fig. 1Bc). Increased miR-137 plasmatic levels were also reversed by MitoQ treatment (Fig. 1Bd; see also Fig. S3 for the other groups). Thus, MitoQ can reverse the OxS-induced miR-137 overexpression, COX6A2 decrease, mitophagy, and PVI impairment, highlighting that the upregulation of miR-137 and subsequent mitophagy defects constitute one of the molecular mechanisms underlying the OxS-induced PVI impairment.

### miR-137 inhibitor injection rescue COX6A2, PVI, and 8-oxo-DG staining in ACC of *Gclm*-*KO*+*GBR* animals

To explore whether miR-137 overexpression led to mitochondrial changes, *Gclm*-KO + GBR mice were injected with miR-137 inhibitor (i-mmu-miR-137; miRCURY inhibitor Qiagen) in ACC of one hemisphere while scramble miR was applied to the contralateral side as control. Inhibition of miR-137 led to decreased oxidative stress marker 8-oxo-DG and increased PV and COX6A2 staining in *Gclm*-KO + GBR as compared to Sham injection (Fig. 1Cc,d). These findings demonstrated a causal mechanistic link between miR-137 overexpression and its targets as hypothesized above.

### Reverse translation: altered plasma exosome miR-137, mitophagy marker, and COX6A2 levels in early psychosis patients

**Fig. 2:**
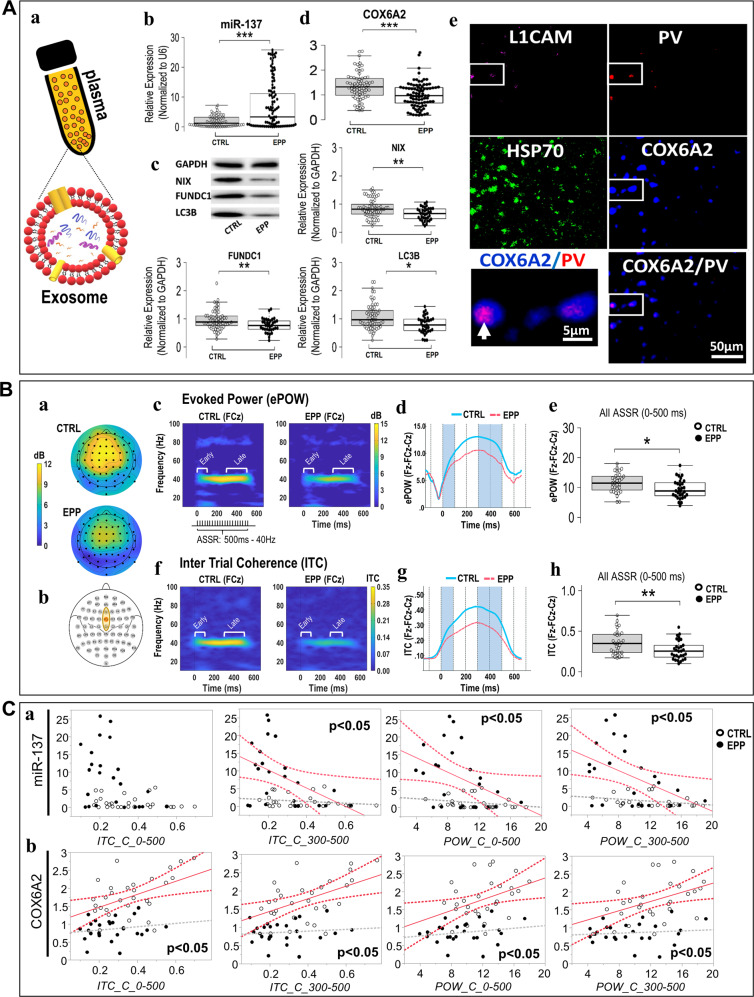
Observations in early psychosis patients (EPP). **A** Alterations in exosomal markers. (**a**) Schema depicting plasma exosomal component. (**b**) Plasma levels of exosomal miR-137 are elevated in EPP subjects as compared to healthy controls. (**c**) Mitophagy markers NIX, FUNDC1 and LC3B decreased in EPP subjects. (**d**) Exosomal plasma COX6A2 levels lower in EPP subjects than in controls. (**e**) Colocalization (arrows; zoomed panel) of COX6A2 and parvalbumin in exosomes of neuronal origin (L1CAM staining). HSP70 staining: exosomal marker (brain and not brain-derived exosomes). *p* < 0.001***, *p* < 0.01**, *p* < 0.05*. **B** Impaired 40-Hz auditory steady-state response in EPP. Topographic maps (a) display the averaged evoked power (ePOW) activity between 38-42 Hz and during the entire ASSR period (0–500 ms) for both CTRL and EPP. Responses depict maximal activities measured at (b) the frontocentral ROI (Fz-FCz-Cz) for ePOW (**c**–**e**) and intertrial phase coherence (ITC; **f**–**h**). Time-frequency maps represent (**c**) ePOW and (**f**) ITC at FCz, where color scales indicate ePOW and ITC amplitude values. Line plots show the time course of (**d**) ePOW and (**g**) ITC values at the frontocentral ROI (averaged activities from Fz-FCz-Cz) from baseline to post-stimulation for EPP (pink) and CTRL (blue). Boxplots report significant (**e**) ePOW and (**h**) ITC differences between CTRL and EPP at the frontocentral ROI for total mean activity (0–500 ms), as well as early- (0–100 ms) and late-latency (300-500 ms) responses (Fig. S4). *p* < 0.01**, *p* < 0.05*, *p* < 0.07^Δ^. C Exosomal levels of miR-137 and COX6A2 as surrogate markers of PV alteration. (**a**) Exosomal miR-137 levels and late-latency gamma oscillations in the frontocentral ROI recording sites are negatively associated in EPP subjects but not in controls. (**b**) Exosomal COX6A2 protein levels associated positively with both late-latency ITC and ePOW in healthy controls but not in EPP (values at frontocentral ROI = averaged activities from Fz-FCz-Cz).

We then assessed plasma miR-137, COX6A2, and mitophagy marker levels in an EPP cohort. Exosomal miR-137 levels were higher in the EPP group than in age- and gender-matched healthy controls (Fig. 2Aa,b). The plasma levels of mitophagy receptors NIX and FUNDC1 as well as of the autophagosome LC3B were lower in the EPP group than in control samples (Fig. 2Ac), suggesting a mitophagy defect in patients. Thus, the OxS-induced upregulation of miR-137 leading to mitophagy marker deficits observed centrally and peripherally in the animal model, were observed peripherally in EPP.

Exosomal COX6A2 levels were lower in the EPP group than in the healthy controls (Fig. 2Ad). Additionally, by surface marker identification through neural-specific antibodies, we highlighted the colocalization of COX6A2 and parvalbumin in exosomes of neuronal origin (using an antibody against neural cell adhesion molecule L1 (L1CAM)) [47] (Fig. 2Ae, arrows; zoomed panel), emphasizing that plasma exosomal COX6A2 levels could reflect central PVI integrity.

### Altered gamma-band auditory steady-state responses (ASSRs) in early psychosis patients

Patients with SZ often exhibit a deficit in the power and/or phase-locking of ASSRs, particularly in the gamma frequency range [2]. In *Gclm*-KO mice, we previously reported an OxS-induced PVI impairment associated with a gamma oscillation deficit [25, 28]. To explore whether PVI circuitry was affected in our EPP cohort, we assessed ASSR power and phase-locking to gamma range stimulation. The intertrial coherence (ITC) and evoked power (ePOW) indices of the ASSR were specifically defined by the mean activity within a region of interest (ROI) encompassing the three frontocentral electrode sites Fz, FCz and Cz, given their maximal responses (Fig. 2Ba,b). Over the entire ASSR period (0–500 ms), but particularly during the late-latency ASSR period (300–500 ms) (Fig. S5), there was a reduction in both ePOW and ITC at 40 Hz in the EPP group compared with controls (Fig. 2Bc–h). The trend effect at 80 Hz observed with ePOW values results directly from the 40 Hz effects (harmonic response).

### Association between miR-137 and COX6A2 exosomal levels with auditory steady-state responses (ASSR) in early psychosis patients

As an increase in miR-137 level is associated with PVIs in *Gclm*-KO mice, based on the hypotheses that the ASSR reflects PVI integrity [48] and that increased plasma miR-137 levels in the EPP group represent a proxy marker of PVI impairment, we tested whether high plasma miR-137 levels could be associated with ASSR deficits. Exosomal plasma levels of miR-137 were negatively correlated with both late-latency ITC and ePOW responses in the frontocentral ROI of the EPP group (Fig. 2Ca). Increased plasma levels of miR-137 in the EPP group may thus reflect an impairment in cortical microcircuits mediating late-latency gamma ASSR deficits. Additionally, exosomal COX6A2 protein levels correlated positively with late-latency ITC and ePOW in healthy controls but not in EPP (values at frontocentral ROI = averaged activities from Fz-FCz-Cz) (Fig. 2Cb), suggesting that, under physiological conditions, a high mitochondrial supply corresponds to improved auditory evoked responses, a relationship disrupted in patients. Furthermore, as described above, the colocalization of COX6A2 and parvalbumin in exosomes of neuronal origin highlights that plasma exosomal COX6A2 levels could reflect central PVI integrity (Fig. 2Ae).

### Combined exosomal levels of COX6A2 and miR-137 allowed patient stratification in subgroups with and without mitochondrial dysfunction

**Fig. 3:**
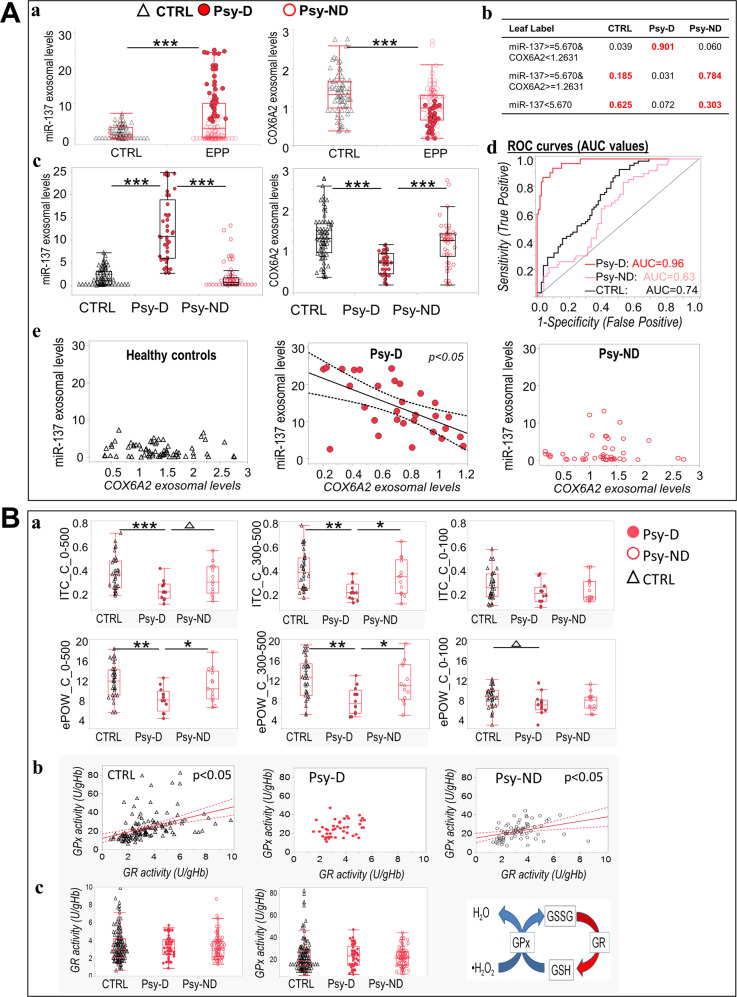
Patient segregation using combined detection of exosomal levels of COX6A2 and miR-137. **A**. (**a**–**c**) Combined use of miR-137 and COX6A2 exosomal levels allows the classification of “Psy-D” psychosis patients with mitochondrial dysfunction and “Psy-ND” with no/low mitochondrial dysfunction, relative to control subjects. (**b**) Response probability of the cohort partition using the cutoff points of the miR-137 and COX6A2 expression levels (for more details, see Fig. S5). (**c**) The plasma levels of exosomal miR-137 and COX6A2 are significantly altered in Psy-D group compared with that in Psy-ND group and in healthy control subjects. (**d**) Based on the combined detection of miR-137 and COX6A2 levels in blood samples, ROC analysis showed an area under the curve (AUC) of 0.96 for the Psy-D group and a smaller AUC for the Psy-ND and control subjects, showing that the combined detection of miR-137 exosomal level and COX6A2 protein level allows a specific identification of Psy-D patients. (**e**) Low exosomal COX6A2 protein levels are associated with high miR-137 exosomal concentrations in the Psy-D group. **B** Impaired ASSR and redox dysregulation in EPP is associated with elevated miR-137 exosomal levels and low exosomal COX6A2 protein levels. (**a**) Both ITC and evoked power were decreased in the Psy-D psychosis patients compared with those in the Psy-ND psychosis subjects and control group. Psy-ND and control groups are at the same level. (**b**) Correlations between GPx and GR activity were only observed in the Psy-ND psychosis subjects and control subjects. (**c**) No difference was observed in the levels of GPx and GR between groups.

Using baseline ranges of exosomal levels of both miR-137 and COX6A2, (Fig. 3Aa) two subgroups of patients were identified with jmp software (Jmp software, bitplane): 1) One having the same range of miR-137 (mean (4.8) ± 2 SD (0.435) = cutoff point) and COX6A2 (mean (1.26) ± 2 SD (0.031)=cutoff point) values as healthy controls, named “Psy-ND” for psychosis patients with no/low mitochondrial dysfunction; 2) the other with values diverging from the controls values, exhibiting a cutoff point higher than 5.67 (mean + 2 SD) for miR-137 and lower than 1.2 (mean-2SD) for COX6A2, named “Psy-D” for psychosis patients with high mitochondrial dysfunction (Fig. 3Ab,c). The COX6A2 and miR-137 cutoff points were validated using a partition algorithm. The generated decision tree confirmed that these cutoff points were optimal for best predicting patient clustering (Fig. 3Ab, Fig. S6). Furthermore, Fig. 3Ad shows the discrimination performance following the combined use of miR-137 and COX6A2 exosomal levels, presented as receiver operating characteristic (ROC) curves. The ROC curve and corresponding area under the curve (AUC) give an overall profile of the cutoff-based clustering. The AUC of Psy-D subjects (AUC = 0.96) was higher than that of both Psy-ND (AUC = 0.63) and control subjects (AUC = 0.74), showing that the combined detection of miR-137 exosomal levels and COX6A2 protein levels allowed an optimal identification of Psy-D patients, with the highest sensitivity and specificity, i.e., determining true positives and distinguishing them from true negatives. Interestingly, these two patient subgroups exhibited different correlation patterns between the levels of miR-137 and COX6A2: high miR-137 exosomal concentrations were associated with low exosomal COX6A2 protein levels in Psy-D but not in Psy-ND or in control subjects (Fig. 3Ae).

### Impaired ASSR and dysregulation of the redox system is associated with the EPP subgroup with mitochondrial dysfunction

Compared with the Psy-ND and control groups, the Psy-D group exhibited deficits in ITC and ePOW (Fig. 3Ba). Psy-D patients almost exclusively exhibited high miR-137 and low COX6A2 plasma levels alongside ASSR deficits. As miR-137 upregulation and COX6A2 down-regulation were observed in transgenic mice exhibiting redox dysregulation (i.e., *Gclm*-KO mice), we wondered whether the GSH redox cycle (blood cell activities of GSH peroxidase [GPx] and GSH reductase [GR]) differed between patient subgroups. We found that both controls and Psy-ND individuals displayed a well-regulated redox balance, as revealed by a positive correlation between GPX and GR (Fig. 3Bb). In contrast, this correlation was absent in the Psy-D group, suggesting that this group was characterized by a dysregulated antioxidant defense and/or OxS (Fig. 3Bb). No significant difference was observed at the level of GPx or GR activity between groups (Fig. 3Bc).

### More severe clinical symptoms and cognitive deficits in the EPP subgroup with mitochondrial dysfunction

**Fig. 4:**
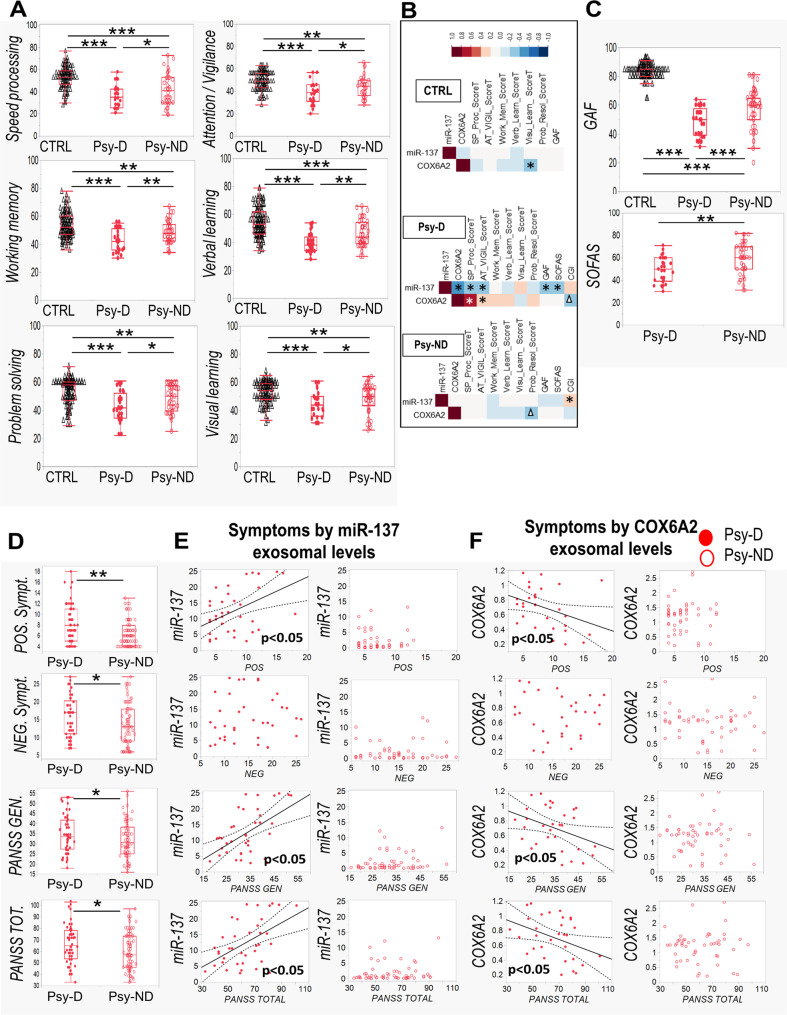
Cognitive deficits and symptom severity in relation to the stratification based on the combined detection of miR-137 and COX6A2 levels. (**A** Boxplots displaying neurocognitive domains (speed processing, attention/vigilance, working memory, problem-solving and verbal and visual learning) according to the stratification Psy-D (with mitochondrial dysfunction) and Psy-ND (with no/low mitochondrial dysfunction). Psy-D subjects exhibit worse performance in cognitive tests than Psy-ND subjects and healthy controls. **B** Correlations between neurocognitive and functional profiles and exosomal levels of miR-137 and COX6A2. **C** Psy-D individuals displayed lower GAF, and SOFAS scores than Psy-ND individuals. **D** Psy-D subjects presented more symptoms on the PANSS scale than Psy-ND psychosis subjects. **E** Exosomal levels of miR-137 in the Psy-D individuals (red dots) positively correlated with symptoms severity while Psy-ND (empty circles) do not. **F** Exosomal levels of COX6A2 in the Psy-D individuals negatively correlated with symptoms severity. *p* < 0.001***, *p* < 0.01**, *p* < 0.05*, *p* < 0.07^Δ^.

To explore the possibility of distinct psychopathological and neurocognitive profiles in relation to the combined miR-137- and COX6A2-based stratification, i.e., to the Psy-D and Psy-ND subgroups, we assessed cognitive functions and clinical symptoms in the patient and control groups. In cognitive tests involving speed processing, attention/vigilance, working memory, problem-solving and verbal and visual learning, the Psy-D subjects performed worse than the Psy-ND and control subjects (Fig. 4A). Specifically, in Psy-D subgroup, high miR-137 levels and low COX6A2 levels were associated with low neurocognitive performance (Fig. 4B). Moreover, in line with the combined miR-137 and COX6A2 stratification, the Psy-D subgroup showed lower Global Assessment Functioning (GAF) and Social and Occupational Functioning Assessment Scale (SOFAS) scores (Fig. 4C) than Psy-ND group. Accordingly, high miR-137 levels were associated with low global and social functioning scores in the GAF and SOFAS, respectively, only in Psy-D subgroup. (Fig. 4B). Compared with those in the Psy-ND subgroup, patients in Psy-D subgroup exhibited more severe positive, negative and general symptoms as assessed by the PANSS (Fig. 4D). In the Psy-D group, higher plasma miR-137 levels and lower COX6A2 levels were associated with more severe symptoms (Fig. 4E, F). Thus, Psy-D patients with mitochondrial dysfunction exhibited a worse psychopathological status, neurocognitive performance and global and social functioning. No difference was observed in CPZ-equivalent dose of antipsychotics between EPP subgroups (Fig. S7, Table S3).

## Discussion

**Fig. 5:**
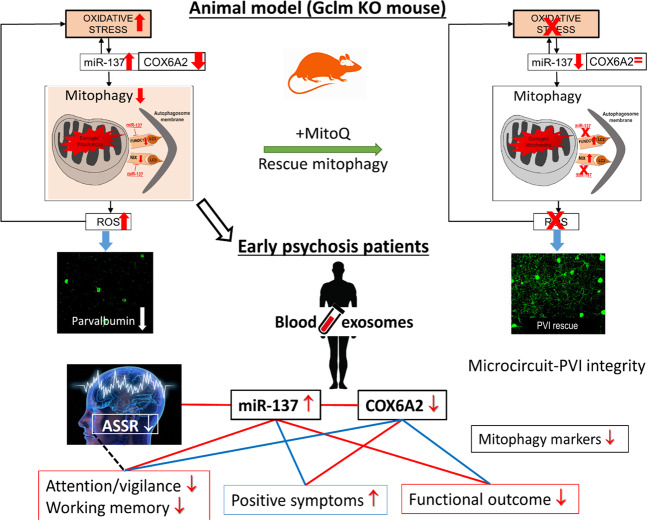
Schematic representation of our reverse translational findings in mice and patients. Reverse translational findings reveal that exosomal mitophagy alterations are linked to oxidative stress-induced impairments of parvalbumin interneuron (PVI)-microcircuit in association with gamma oscillation alterations and cognitive deficit. In prefrontal cortex and in blood of redox dysregulated mice (Gclm-KO + GBR), oxidative stress induces miR-137 upregulation, leading to decreased COX6A2 and mitophagy markers, including NIX, Fundc1 and LC3B, and to accumulation of damaged mitochondria. This would further exacerbate oxidative stress and PVI impairments in a positive feedforward process. MitoQ, a mitochondria-targeted antioxidant, rescued all these processes. Translating to early psychosis patients, blood exosomal miR-137 increases and COX6A2 decreases, combined with mitophagy markers alterations suggest that observations made centrally and peripherally in the animal model were reflected in patients’ blood. Higher exosomal miR-137 and lower COX6A2 levels were also associated with a reduction of ASSR gamma oscillations in EEG. As ASSR requires proper PVI-related networks, alterations in combined miR-137/COX6A2 plasma exosome levels may represent a proxy marker of PVI cortical microcircuit impairment that are critically involved in SZ psychopathology and cognition. Blue line: positive correlations; red line: negative correlations; black dashed line: loss/gain association.

Oxidative stress is recognized as a central pathological feature of SZ. Herein, we report that in prefrontal PVIs of redox dysregulated mice (*Gclm*-KO + GBR), OxS induces miR-137 upregulation, leading to decreased COX6A2 in PVIs and to mitophagy and accumulation of damaged mitochondria, further exacerbating OxS and PVI impairment. MitoQ, a mitochondria-targeted antioxidant, rescued all these processes. Translating to EPP patients, blood exosomal miR-137 increases and COX6A2 decreases, combined with mitophagy markers alterations, suggest that observations made centrally in our animal model were reflected peripherally in EPP. Higher exosomal miR-137 and lower COX6A2 levels were also associated with a reduction of ASSR gamma oscillations in EEG. As ASSR requires proper PVI-related networks [49], alterations in combined miR-137/COX6A2 plasma exosomal levels may represent a proxy marker of PVI cortical microcircuit impairment that are critically involved in SZ psychopathology and cognition. These findings allowed stratifying EPP into two subgroups: (a) a “Psy-D” subgroup, characterized by high miR-137 and low COX6A2 levels in exosomes, presumably representing the dysfunctional mitochondria in PVIs, and (b) a “Psy-ND” subgroup, including patients having miR-137 and COX6A2 levels in the same ranges as controls. Psy-D patients exhibited more impaired ASSR responses in association with worse psychopathological status, neurocognitive performance, and global and social functioning, suggesting that impairment of PVI mitochondria would lead to a more severe disease profile (see summary Fig. 5).

Here, we provide preclinical evidence that OxS induces a remarkable and specific elevation of miR-137 associated with decreased COX6A2, colocalized with parvalbumin in the prefrontal PVI cell bodies as well as in processes including spines and terminals. Inhibition of miR-137 reversed the oxidative stress, as well as alterations of PVI and COX6A2 (Fig. 1Cc,d), thus indicating that miR-137 is causal to its downstream targets and its ensuing vicious cycle (Fig. 5). Of note, COX6A2, a subunit of the terminal enzyme of the mitochondrial respiratory chain cytochrome c oxidase COX, through coupling the electron transfer from cytochrome c to molecular oxygen, plays a critical role in ATP synthesis by creating a proton electrochemical gradient across the inner mitochondrial membrane. Interestingly, COX6A2 is selectively expressed in highly active cardiac and skeletal muscles [38], its expression in fast-spiking PVIs would be consistent with their intense oxidative metabolism [50].

Overexpression of miR-137 triggers a cascade of molecular alterations (e.g., Nix, LC3B, and Fundc1), leading to ultrastructural fragmentation and densification of mitochondria that culminates in aberrant mitophagy. Consequently, a feedback process may ensue in which damaged mitochondria generate ROS, causing additional OxS in PVIs and driving a vicious circle of OxS within these cells (see Fig. 5, upper panel). This may potentially occur in a manner similar to the reported vicious circles resulting from feedforward reciprocal interactions between redox dysregulation, NMDAR hypofunction, and neuroimmune dysfunction [8, 51].

MitoQ reversed the cell morphology and molecular abnormalities observed in the prefrontal PVIs of *Gclm*-KO mice is consistent with the critical role of mitochondrial dynamics, biogenesis, and turnover known to be vulnerable to OxS in dendritic arbor morphology [52, 53] and treatment with mitochondria-targeted antioxidants would represent a key strategy in preventing long-term alterations to PVIs. MitoQ is a targeted synthetic analog of the reduced form of coenzyme-Q10, a non-target-specific antioxidant. MitoQ is able to cross the blood-brain barrier, as well as plasma and mitochondria membranes thanks to its lipophilic triphenyl-phosphonium-cation [54]. In Parkinson disease, MitoQ has proved to reduce neurotoxicity in a preclinical model [6], but has failed to show any effect on the disease progression in a 1-year double blind placebo-controlled trial in patients [55]. To our knowledge, no studies have investigated the effect of MitoQ in psychiatric disorders.

The translational observations made in a cohort of patients in the EPP revealed elevated miR-137 and decreased COX6A2 and mitophagy marker levels in plasma exosomes (Fig. 2A), changes similar to those measured in the ACC of *Gclm*-KO mice exposed to persisting oxidative stress (Fig. 1). Importantly, the observation that, in *Gclm*-KO + GBR model, miR-137 plasma levels were increased (Fig. 1Bd) and correlated positively with its prefrontal levels (Fig. 1Be) suggest that elevated miR-137 exosomal plasma levels in patients (Fig. 2Aa, Fig. 3Aa) may reflect their central levels. Genetic and/or environmental risk factors may be at the origin of miR-137 overexpression in patients, and the ensuing feedforward vicious cycle of oxidative stress may represent the underlying mechanism (Fig. 5, upper panel). Such interpretation is in line with the observed balanced redox regulation (positive correlation between GPx and GR blood cells activities), present in control and Psy-ND subjects but disrupted in Psy-D subjects (Fig. 3Bb). Interestingly, the miR-137 single nucleotide polymorphism (SNP) rs1625579 was one of the best-replicated, genome-wide significant loci strongly associated with SZ [37, 56], thus warranting further investigations. The translation from mouse model to patients and the differential classification between Psy-D and Psy-ND subgroups was made possible with development of state-of-the-art brain-derived exosomes that could be accessed through peripheral fluid such as plasma [57, 58], thus allowing peripheral insight into neural state. More importantly, COX6A2 and parvalbumin appeared to be colocalized in exosomes of neuronal origin, as indicated by L1CAM staining. The analysis of exosomal content warrants the identification of blood-based biomarkers in early psychosis. While, ASSR gamma oscillations of Psy-ND patients are identical to those of controls, Psy-D patients presented a decreased oscillations power, suggesting that miR-137 and COX6A2 alterations mirror cortical PVI microcircuit impairments. The increased level of miR-137 together with the decreased level of COX6A2 in exosomes, particularly in association with gamma oscillations in EEG responses, could thus reflect their alteration in the central nervous system, likely in PVIs, as demonstrated in the animal model, although the contribution of other, subcortical, PV neurons can not be excluded.

We highlighted for the first time the combined profile of exosomal miR-137 and COX6A2 as two novel biomarkers, most likely reflecting in plasma the state of PVI mitochondria. This allows to distinguish two subgroups of patients: the Psy-D group, with high miR-137 and low COX6A2 levels, and the Psy-ND group, with miR-137 and COX6A2 levels similar to control values (Fig. 3Ac). In the ROC curve analysis, an AUC of 0.96 for Psy-D versus an AUC of 0.63 for the Psy-ND group highlighted a powerful performance of the discrimination with one of the highest sensitivity and specificity values reported in the field of psychiatry. In the Psy-D patients, the negative correlation between miR-137 and COX6A2 levels indicated that the mitochondrial damage was proportional to the overexpression of miR-137 (Fig. 3Ae). It is important to note that the Psy-D patients had a larger deficit in ASSR gamma oscillations (Fig. 3Ba), more cognitive deficits and higher PANSS scores (Fig. 4) than the Psy-ND patients. In particular, the PANSS scores correlated positively with miR-137 levels and negatively with COX6A2 levels, indicating a direct relationship between the markers levels and the severity of the symptoms. Although, one cannot exclude the potential contribution of other not assessed factors to this association in patients with most severe symptoms, our results highlight that impairments in the PVI microcircuitry would lead to a more severe disease phenotype.

Remarkably, EPP patients with high plasma levels of miR-137 have impaired 40-Hz ASSRs. In ASSR studies related to the analysis of brain oscillations, two main parameters are typically analyzed: the intertrial coherence (ITC), and the evoked power (ePOW),both typically affected in SZ and EPP patients [40, 59]. Late-latency ITC and ePOW were negatively correlated with plasma exosome levels of miR-137 in the EPP group (Fig. 2Ca). This observation may reflect cortical microcircuit deficits stemming from central and left lateralized gamma ASSR alterations in these individuals. On the other hand, late-latency ITC and ePOW were positively correlated with exosome COX6A2 levels in healthy controls, emphasizing the contribution of the mitochondria to the efficiency of PVIs in the maintenance of gamma oscillations (Fig. 2Cb).

Our study had a noteworthy limitation. Most of the individuals in the EPP group were undergoing antipsychotic treatment (Tables S4 and S5); however, this treatment was minimal as they were recruited at the early stages of psychosis. More research studies are warranted with larger numbers of unmedicated patients to validate and extend the current findings.

Early detection and intervention could improve clinical and functional outcomes in schizophrenia [60] or even prevent the transition to psychosis. The challenge remains however the patients’ heterogeneity at the levels of genetics, pathophysiology and clinical phenomenology. The identification of mechanism-based, reliable translational biomarkers allowing better stratification of patients, personalization of treatment and monitoring of disease progression remain an unmet need. Our results revealed that the miR-137 and COX6A2 exosomal levels from a relatively substantial sample size (*n* = 272) were altered in association with decreases in the levels of mitophagy markers in blood samples collected from the EPP group. The plasma levels of combined exosomal miR-137 and COX6A2 may represent mechanism-based proxy biomarkers of PV neurons impairment, potentially useful for the selection of patients, monitoring of the disease evolution and, in clinical trials, assessment of treatment efficacy. Furthermore, alterations in the subcomponents of the ASSR gamma oscillations, together with combined miR-137 and COX6A2 indices, could represent potential biomarkers of PVI impairment in early psychosis, useful for biomarker-guided treatment targeting PVIs mitochondrial impairment in specific subgroups of patients, particularly Psy-D subgroup. Indeed, this would allow the monitoring an intervention’s impact relying on both peripheral and central markers at the individual level. Taken together, our findings revealed pathophysiological, mechanism-based, reliable biomarkers leading to the selection of homogenous patient subgroups and objective measures to capture clinical efficacy in novel drug trials. From a translational standpoint, future stratified clinical trials assessing the effect of mitochondria-targeted antioxidants in biomarker-guided treatment of psychosis and SZ would be warranted. Given the safety profile of mitochondria-targeted antioxidants, our results pave the way for the much-needed precision diagnosis and early treatment at the individual level for the very heterogeneous “clinical high risk” or “at risk mental state” subjects, eventually leading to a better prognosis and prevention of serious mental disorders [61, 62].

In addition to psychotic disorders and SZ, the proposed mechanism-based biomarker profile, which could lead to stratified clinical trials with mitochondria-targeted antioxidants, may also be applied to other brain diseases. Indeed, OxS, and impairments in the mitochondria, excitatory-inhibitory balance, neural synchronization, and cognition have been linked to other major psychiatric and neurological disorders, including autism [63], bipolar disorder [64], mood disorders [65], anxiety disorders [66], epilepsy [67], age-associated diseases [68, 69], and cognitive impairment [70].

## Supplementary information

Supplementary Materials, subjects and methods

Figure and supplementary figure and table Legends

Supplementary Figure 1

Supplementary Figure 2

Supplementary Figure 3

Supplementary Figure 4

Supplementary Figure 5

Supplementary Figure 6

Supplementary Figure 7

Supplementary Table 1

Supplementary Table 2

Supplementary Table 3

Supplementary Table 4

Supplementary Table 5

Supplementary Table 6

## Data Availability

Further information and request for resources and reagent should be directed to and will be fulfilled by the Lead Contact, Kim Q Do (Kim.do@chuv.ch).
