# Supplementary material for: Mitochondrial, exosomal miR137-COX6A2 and gamma synchrony as biomarkers of parvalbumin interneurons, psychopathology, and neurocognition in schizophrenia
Source: Mol Psychiatry. 2021 Oct 22;27(2):1192–204. doi: 10.1038/s41380-021-01313-9 (PMC9054672; doi:10.1038/s41380-021-01313-9)
Supplement: Supplementary file 1 — Supplementary Materials, subjects and methods [file 41380_2021_1313_MOESM1_ESM.docx]

**Supplementary Materials, subjects and methods**

**PRECLINICAL, MICE STUDY**.

Transgenic mice lacking the glutamate cysteine ligase modifier subunit (*Gclm*-KO; B6.129-*Gclm* tm1Tdal, a generous gift from Dr. T. Dalton, University of Cincinnati, USA) were crossbred with C57BL/6J mice over more than 10 generations(1). The mice were housed under a 12-hour light-dark cycle in groups of 3–5 per cage. All experiments were performed using female and male animals (Fig. 1C). The mice had access to food and water or a MitoQ solution.

**Tissue preparation**. The mice were anesthetized with a lethal dose of sodium pentobarbital (60 mg/kg, intraperitoneal), transcardially perfused with a paraformaldehyde solution (PFA, Electron Microscopy Sciences, Hatfield) and postfixed in the same solution at room temperature (RT). Dissected brains were cryoprotected using a 30% sucrose solution. Coronal cryosections (50 µm) of the anterior cingulate cortex (ACC) were obtained on a freezing microtome.

**Immunohistochemistry**. Several sections per animal were selected where it was possible to clearly visualize the ACC. These sections were rinsed three times with PBS 0.1 M, pH 7.4. Subsequently, the free-floating sections were fixed in 4% PFA diluted in PBS 0.1 M to decrease background signals stemming from proteinase K (proteinase K at 1 µg/ml in PBS 0.1 M). The sections were then incubated for 1 hr at RT in a blocking solution (BS) consisting of PBS 0.1 M with 0.1% bovine serum albumin (BSA) and 0.1% Tween. Then, the tissue was incubated overnight at 4°C with primary antibodies against BNIP3L or NIX (NIP3-like protein X) (mouse, Cell Signaling Technology, 1:500), PV (sheep, R&D Systems, 1:2500), LC3B (rabbit, Cell Signaling Technology, 1:500), 8-oxo-dG (mouse, TREVIGEN, 1:300), and FUN14 Domain Containing 1 (FUNDC1) (rabbit, Abcam, 1:500). While BNIP3L and FUNDC1 are mitophagy receptors, LC3B is an autophagosome receptor, and each of these proteins directly contributes to mitophagy(2). Additionally, 8-oxo-dG is a DNA adduct formed by the reaction of hydroxyl radicals with the DNA base guanine(3) and is used to visualize OxS(4). The following day, the tissue was rinsed five times with PBS 0.1 M, pH 7.4 (30 minutes per wash) and PBST buffer (PBS 0.1 M and 0.1% Tween). Following these steps, the samples were incubated in secondary antibody coupled with fluorophore (Alexa Fluor 594 chicken anti-mouse (Invitrogen), Alexa Fluor 488 donkey anti-sheep (Invitrogen) and Alexa Fluor 647 goat anti-rabbit (Invitrogen)) for 1.5 hrs. Washes were then performed twice in PBST buffer (once at 5 minutes and once at 10 minutes) and with Phosphate Buffer (0.1 M phosphate buffer, pH 7.4). Subsequently, 4′,6-diamidino-2-phenylindole (DAPI) solution (1 mg/ml of PBS 0.1 M, pH 7.4) was applied to the sections for 10 min. The final washes were performed in PB (twice for 5 minutes), and the samples were mounted on 4% gelatin-coated slides with Mowiol 4-88 (Calbiochem, La Jolla, CA).

**Imaging and Image Processing**. Z-stack images were acquired using a Zeiss AxioObserver Microsystems confocal microscope with a Plan-Neofluar 40x/1.30 NA objective. Staining patterns of all images were processed and quantified using Imaris software. For cell count, to ensure that our counting takes careful consideration of the variation in shape and size of cells, our cell count criterion was set to include profiles with soma size (from 5 - 12 µm) and normalized to total cell number (DAPI staining).

For co-localization analysis, in addition to the high-resolution Z-stack image acquisition, we processed all Z-stack with Huygens Deconvolution Software that correct the artifact to give us the co-localization coefficients. We also double validate analysis using Colocalization Analyzer module to obtain all needed information concerning the amount of spatial overlap between the two studied channels.

**MicroRNA *in situ* hybridization**. We performed microRNA *in situ* hybridization on the same mouse brain sections that had previously been used for immunostaining purposes. Following initial washing in PBS 0.1 M (pH 7.4), each section was treated with proteinase K solution (proteinase K at 1 µg/ml in PBS 0.1 M) at 37°C for 10 min. Subsequently, an acetylation step involving the incubation of sections for 10 minutes in a pH 8 solution containing 0.5% acetic anhydride, 1.35% triethanolamine, and 0.067% HCl in 0.1% DEPC-treated water was performed to reduce any nonspecific binding. The miRNA detection probes (miRCURYTM LNA detection probes, Exiqon) were then diluted in hybridization buffer (formamide, SSC, yeast RNA, heparin, Denhardt’s solution, Tween, EDTA), and the slides were incubated in 300 μl of the hybridization solution, covered with coverslips and sealed. The slides were then incubated for 2 minutes at 90°C to denature the dsRNA and cooled on ice. The sections were then incubated with the probes at 50°C overnight in a wet chamber humidified with a 50% formamide-5X SSC (sodium chloride, sodium citrate) solution. The following day, each section was rinsed with 5X SSC solution and developed at RT in the dark until a red precipitate started to appear. To stop the color reaction, the sections were washed with PBS 0.1 M and PB at RT before being mounted on gelatin-coated slides with Mowiol.

**Electron Microscopy: Fixation, Embedding, and Image Acquisition**. Mice were anesthetized with sodium pentobarbital (60 mg/kg, i.p.) and transcardially perfused with PFA and glutaraldehyde in phosphate buffer. Then, the brains were removed, and 60 μm coronal sections were collected from the prefrontal cortex with a vibratome. These sections were washed in cacodylate buffer, postfixed in 1% osmium tetroxide in 0.1 M cacodylate buffer, dehydrated in alcohol and propylene oxide, and embedded between silicon-coated glass slides in Durcupan resin. Once the resin had cured, we identified the ACC to prepare the block, including this specific region. A series of 50 nm sections were cut and collected on single-slot grids bearing a Formvar support film. Finally, sections were washed in double distilled water and contrasted with lead citrate.

Serial images of the neuropil in layers 2 to 5 were collected using a CCD camera and iTEM software (Olympus, Germany) on a Philips CM10 electron microscope, aligned and analyzed using Fiji software.

**Quantification of plasmatic miR-137 in mouse.** miR-137-3p miREIA (BioVendor) measurement was developed in collaboration with Dr. Jochen Hauer (BioVendor GmbH). Calibration curves were constructed according to the product data sheet. Hybridization was done in peqStar thermocycler (Peqlab). After incubation of all reactions, absorbance was measured in a 96-well reader Infinite M200 (Tecan). Sample concentrations were calculated using Myassays Analysis software.

**HUMAN CLINICAL STUDY**.

**Subjects recruitment**: The study population included early psychosis patients (EPP; *n*=138) and healthy controls (*n*=134), matched for gender and age (Table S1). The patients were recruited from the Treatment and Early Intervention in Psychosis Program (TIPP)(5), which is a specialized 3-year program for the treatment of early psychosis patients.

**Inclusion criteria** were as follows: (i) age between 18 and 35 years old; (ii) residence in Lausanne or the surrounding areas; (iii) meeting of the threshold criteria for psychosis, as defined by the ‘Psychosis threshold’ subscale of the Comprehensive Assessment of At Risk Mental State (CAARMS)(6); (iv) no more than 6 months of treatment with antipsychotic medication for psychosis; (v) no psychosis related to intoxication or organic brain disease; (vi) intelligence quotient ≥70; and (vii) ability to discern and to provide informed consent. The psychosis threshold and the diagnosis assessment resulted from expert consensus. The duration of the illness considered the time elapsed from the psychosis threshold to participation in the study, and the diagnosis was based on DSM-IV criteria (APA, 1994). Most of the patients (*n*=123) were taking antipsychotic medication (374.2±216.5 mg chlorpromazine equivalent dose (CPZ))(7); a detailed list of antipsychotics is found in Table S4).. It is noteworthy that patients who agreed to participate in the present study were representative of the entire clinical TIPP cohort(8). Healthy controls were recruited within the same catchment area(5). They were assessed by the Diagnostic Interview for Genetic Studies(9) to confirm the absence of any major psychiatric or substance use disorders. In addition, healthy controls who reported having a first-degree relative with psychotic disorder were excluded. Neurological disorders and severe head trauma were also exclusion criteria for all subjects.

**Clinical assessment**: Patient symptomatology was evaluated using the Positive and Negative Syndrome Scale (PANSS)(10). The Wallwork/Fortgang five-factor model(11) was used to categorize the different symptom domains, as it better accounts for the dimensional structure of the PANSS compared to the traditional three subscales. This model comprises positive, negative, disorganized/concrete, excited, and depressed factors and has been shown to be differentially affected by various types of traumatic experiences(12). For all participants, the GAF (APA, 1994), a neuropsychological evaluation (MATRICS Consensus Cognitive Battery(13, 14)), blood sampling and EEG recordings were retrieved in a concomitant manner. All MATRICS tests were administered except the Mayer-Salovey-Caruso Emotional Intelligence Test (MSCEIT), given that a validation of the French version was not available at the time of the study. Six cognitive domain scores (normalized for sex and age) were obtained: processing speed, sustained attention, working memory, visual learning, verbal learning and problem solving.

**Blood markers analysis**

***Blood collection and redox marker processing***. Blood was collected in EDTA tubes after overnight fasting (from midnight until blood collection). Whole blood was immediately frozen at -80° to subsequently assess GSH levels as previously described(15). In parallel, the blood was immediately centrifuged at 3,000 g for 5 minutes at 4°C. The pellet, containing only blood cells, was washed 2 times with 0.9% NaCl and frozen at -80°C to subsequently assess glutathione peroxidase (GPx) and glutathione reductase (Gr) activity as previously described(12).

***Quantification of circulating miR-137 (ExomiRs)***.

*miRNA extraction*: miRNAs were extracted from exosomes using a miRNeasy Kit (Qiagen, Hilden, Germany). The protocol used was provided with the kit and described in the “miRNeasy Handbook”. The quality of extracted miRNAs and their concentrations were determined with a NanoDrop (ND-1000 spectrophotometer, Thermo Fisher Scientific, USA) by measuring the absorbance at 260 nm (A260) and 280 nm (A280). An A260/A280 ratio of ~2.0 indicated pure miRNA (as well as pure RNA).

*Reverse transcription:* For first-strand cDNA synthesis, total purified miRNA samples were diluted to 5 ng/µl. miRNA was reverse transcribed using a miRCURY LNA Universal cDNA Synthesis kit (Exiqon, Vedbaek, Denmark) according to the instructions enclosed. A mixture containing 4 μl of total miRNA, 2 μl of the enzyme mix, and 4 μl of 5X reaction buffer was supplemented with nuclease-free water to yield a final volume of 20 μl. The cocktail was gently vortexed to thoroughly mix all reagents. The final solution was spun down and incubated for reverse transcription at 42°C for 60 minutes, followed by 5 minutes at 95°C. The obtained cDNA templates were immediately cooled on ice and stored at 4°C.

*qPCR amplification:* We used cDNA templates and Exiqon master mix (Exiqon, Vedbaek, Denmark) following the product user instructions. This procedure suggests the use of three replicates for each plasma sample and for each miRNA. For amplification, final reaction volumes of 10 μl were prepared with 5 μl of master mix, 1 μl of LNA primer set (0.5 µl each of forward and reverse primers) and 4 μl of 1.25% solution of the cDNA template in nuclease-free water. qPCR amplification was performed with a qPCR Detection System with the following thermal cycling parameters: 10 minutes at 95°C, 50 cycles (10 sec each) at 95°C and 60°C for one minute. The qPCR values (quantification cycle “Cq“) for the studied miRNAs were normalized to three reference miRNAs (miR-Ref), miR-16, snRNA-U1 and snRNA-U6.

***Quantification of plasma levels of mitophagy markers using Western blot***. Total proteins were loaded on 12.5% acrylamide gels for SDS-PAGE and transferred to nitrocellulose membranes. The membranes were blocked in Tris-buffered saline with 2% nonfat dried milk and hybridized overnight at 4°C with a primary antibody diluted in blocking buffer (anti-NIX, Life Technologies 39-3300, anti-FUNDC1, Abcam ab224722 and anti-LC3B GeneTex GT6312). Proteins on immunoblots were revealed using the appropriate secondary antibody conjugated to horseradish peroxidase (HRP) corresponding to the primary antibody (anti-rabbit). Images were acquired and quantified using a Fusion Imaging System (Fusion Solo-S).

***Quantitative determination of the exosome levels of COX6A2*.** An ELISA Kit for COX6A2 detection was used according to the provided instructions (Human cytochrome c oxidase subunit 6A2, mitochondrial (COX6A2) ELISA Kit). Briefly, the assay sample and buffer were incubated in a precoated plate together with COX6A2-HRP conjugate for one hour. After incubation and several washes, the color intensity was measured using a Tecan spectrophotometer (Vitaris AG). A standard curve was plotted relating the intensity of the color to the concentration of the standards. This standard curve was used to define the COX6A2 level in each sample.

**Electroencephalography study**

A subsample of the above-described cohort participated in an EEG study (Table S1). Stimulus presentation and EEG acquisition were performed in 33 patients and 33 matched healthy controls. Two patient and two control datasets were excluded due to excessive artifacts, and one additional patient dataset was excluded for clinical reasons. Important comorbidities have recently been observed, including attention-deficit/hyperactivity disorder (ADHD) and the abuse of a variety of substances. A handful of studies have explored oscillatory brain responses in ADHD, reporting response alterations in these individuals (16, 17). Finally, the datasets from 30 patients and 31 controls were included (Table S1). All participants reported having normal hearing, and almost all of them were right-handed.

***Procedure and auditory stimuli***. EEG recordings were acquired in a semidark and sound-attenuated room. While the subjects watched a silent movie, auditory steady-state stimuli were presented passively and binaurally by means of insert earphones (Etymotic, model ER-4S; http://www.etymotic.com). Stimulus presentation and response recordings were controlled by E-Prime 2.0 (Psychology Software Tools Inc., Pittsburgh, USA; https://pstnet.com/). Steady-state stimuli consisted of 500 ms trains of 1 ms white noise clicks delivered at 40 Hz and at an intensity of 75 dB SPL. The total recording included 150 click trains with 1100 ms intervals.

***Electroencephalography acquisition and preprocessing***. EEGs were continuously recorded from 64 Ag/AgCl electrode sites (BioSemi Active-Two, V.O.F., Netherlands) equally distributed over the scalp according to the 10/20 system(18). The EEGs were digitized at a sampling rate of 1024 Hz with the internal CMS/DRL loop as reference (www.biosemi.com). Impedances were kept below 20 kΩ. Offline preprocessing was performed with EEGLAB (version 14.1.1; Delorme & Makeig, 2004). Data were rereferenced to TP7-TP8 (a mastoid-like reference). Prior to epoching, a 0.1 Hz highpass cutoff was applied to correct for baseline drift, skin potentials and slow artifacts (zero-phase shift Hamming-windowed sinc FIR (finite impulse response) filter; half-amplitude (−6 dB) cutoff; 0.1 Hz transition bandwidth). Channels exhibiting substantial noise were interpolated using 3D spherical spline interpolation(19). No difference was reported in the total number of interpolated channels between groups (3.8 ± 2.0 for EPP patients, 2.9 ± 2.3 for controls). Data were segmented from -500 to 1000 ms according to stimulus onset and then baseline corrected.

Independent component analysis (ICA) was used to correct for ocular, muscle, cardiac, and line noise and other sources of transient noise (Infomax ICA, runica algorithm). Topographic, temporal, and spectral signatures were used to identify artifacts(20, 21). Bad epochs were removed according to the following criteria: >±100 μV change in one time point and amplitude range within an epoch exceeding 200 μV (R^2^=0.3). There was no difference in the final total number of artifact-free epochs (130.7±9.9 for EPP patients, 132.3±8.7 for controls).

***Time-frequency decomposition and statistical analyses***. Time-frequency decompositions of the ASSR were processed within EEGLAB (version 14.1.1(22)). ITC and ePOW were computed, where ITC measures the phase consistency across single trials and ePOW measures the power amplitude of the average evoked potential. Time-frequency decompositions of the ASSR were analyzed using Morlet waveform transforms (f_0_/σ_f_=[6 0.5], 6-cycle wavelet with a slow linear increase (coefficient 0.5), allowing improved frequency resolution at higher frequencies (22)) and applied to each time point of the resulting single artifact-free epochs for ITC and to the event-related potential for ePOW. A 1 Hz step decomposition from 10 to 100 Hz was performed, and baseline (-100 to 0 ms) normalization was applied.

The average gamma activity (ITC and ePOW) between 38 and 42 Hz was calculated at the frontocentral electrode sites (mean ROI defined by Fz, FCz, and Cz), given their maximal responses. Early- and late-latency–specific responses were extracted, as defined by the mean activity among the first 100 ms from stimulus onset and the last 200 ms of the ASSR (300 to 500 ms), respectively.

**Quantification and statistical analysis.** Statistical analyses were performed using JMP software (JMP IN, Version 12.1, SAS, USA). All of the variables assessed passed the Shapiro-Wilk, indicating that they had a normal distribution. In our analysis, the least squares method revealed no interactions between the variables of interest and age or sex, which were therefore not included in our models.

For correlation analysis, we used the Pearson correlation coefficient to investigate the correlation between variables of interest. Computations of Z-scores after using Fisher’s Z-transformation were carried out for all Pairwise combinations to see whether the change in correlation coefficient is statistically significant between analyzed groups (WT-Gclm vs KO-Gclm / EPP vs. CTRL / Psy-D vs Psy-ND patients). (see supplementary table 6).

For group comparisons, we performed one-way ANOVA followed by Student’s t-test for post hoc comparisons. In all analysis we tested the significance of type I error (with p – value < 0.05* / <0.01** / < 0,001***) for each boxplot using the t-test followed by the estimation of the type II error to verify the power by which the null hypothesis was rejected. Adjustments for multiple comparisons were applied when required.

**References**

1. Steullet P, Cabungcal JH, Bukhari SA, Ardelt MI, Pantazopoulos H, Hamati F, et al. The thalamic reticular nucleus in schizophrenia and bipolar disorder: role of parvalbumin-expressing neuron networks and oxidative stress. Mol Psychiatry. 2018;23(10):2057-65.

2. Narendra DP, Youle RJ. Targeting Mitochondrial Dysfunction: Role for PINK1 and Parkin in Mitochondrial Quality Control. Antioxid Redox Sign. 2011;14(10):1929-38.

3. KamathLoeb AS, Hizi A, Kasai H, Loeb LA. Incorporation of the guanosine triphosphate analogs 8-oxo-dGTP and 8-NH2-dGTP by reverse transcriptases and mammalian DNA polymerases. Journal of Biological Chemistry. 1997;272(9):5892-8.

4. Cabungcal JH, Counotte DS, Lewis E, Tejeda HA, Piantadosi P, Pollock C, et al. Juvenile antioxidant treatment prevents adult deficits in a developmental model of schizophrenia. Neuron. 2014;83(5):1073-84.

5. Baumann PS, Crespi S, Marion-Veyron R, Solida A, Thonney J, Favrod J, et al. Treatment and early intervention in psychosis program (TIPP-Lausanne): Implementation of an early intervention programme for psychosis in Switzerland. Early Interv Psychiatry. 2013;7(3):322-8.

6. Yung AR, Yuen HP, McGorry PD, Phillips LJ, Kelly D, Dell'Olio M, et al. Mapping the onset of psychosis: the Comprehensive Assessment of At-Risk Mental States. Aust N Z J Psychiatry. 2005;39(11-12):964-71.

7. Andreasen NC, Pressler M, Nopoulos P, Miller D, Ho BC. Antipsychotic dose equivalents and dose-years: a standardized method for comparing exposure to different drugs. Biol Psychiatry. 2010;67(3):255-62.

8. Golay P, Baumann PS, Jenni R, Do KQ, Conus P. Patients participating to neurobiological research in early psychosis: A selected subgroup? Schizophr Res. 2018;201:249-53.

9. Preisig M, Fenton BT, Matthey ML, Berney A, Ferrero F. Diagnostic interview for genetic studies (DIGS): inter-rater and test-retest reliability of the French version. Eur Arch Psychiatry Clin Neurosci. 1999;249(4):174-9.

10. Kay SR, Fiszbein A, Opler LA. The positive and negative syndrome scale (PANSS) for schizophrenia. Schizophr Bull. 1987;13(2):261-76.

11. Wallwork RS, Fortgang R, Hashimoto R, Weinberger DR, Dickinson D. Searching for a consensus five-factor model of the Positive and Negative Syndrome Scale for schizophrenia. Schizophrenia Research. 2012;137(1-3):246-50.

12. Alameda L, Fournier M, Khadimallah I, Griffa A, Cleusix M, Jenni R, et al. Redox dysregulation as a link between childhood trauma and psychopathological and neurocognitive profile in patients with early psychosis. Proc Natl Acad Sci U S A. 2018;115(49):12495-500.

13. Kern RS, Nuechterlein KH, Green MF, Laade LE, Fenton WS, Gold JM, et al. The MATRICS consensus cognitive battery, part 2: Co-norming and standardization. Am J Psychiat. 2008;165(2):214-20.

14. Nuechterlein KH, Green MF, Kern RS, Baade LE, Barch DM, Cohen JD, et al. The MATRICS consensus cognitive battery, part 1: Test selection, reliability, and validity. Am J Psychiat. 2008;165(2):203-13.

15. Gysin R, Kraftsik R, Boulat O, Bovet P, Conus P, Comte-Krieger E, et al. Genetic dysregulation of glutathione synthesis predicts alteration of plasma thiol redox status in schizophrenia. Antioxid Redox Signal. 2011;15(7):2003-10.

16. Başar E, Başar-Eroğlu C, Güntekin B, Yener GG. Brain's alpha, beta, gamma, delta, and theta oscillations in neuropsychiatric diseases: proposal for biomarker strategies. Suppl Clin Neurophysiol. 2013;62:19-54.

17. Khaleghi A, Zarafshan H, Mohammadi MR. Visual and auditory steady-state responses in attention-deficit/hyperactivity disorder. Eur Arch Psychiatry Clin Neurosci. 2018.

18. American Electroencephalographic Society guidelines for standard electrode position nomenclature. J Clin Neurophysiol. 1991;8(2):200-2.

19. Perrin F, Pernier J, Bertrand O, Echallier JF. Spherical splines for scalp potential and current density mapping. Electroencephalogr Clin Neurophysiol. 1989;72(2):184-7.

20. Delorme A, Sejnowski T, Makeig S. Enhanced detection of artifacts in EEG data using higher-order statistics and independent component analysis. Neuroimage. 2007;34(4):1443-9.

21. Jung TP, Makeig S, Humphries C, Lee TW, McKeown MJ, Iragui V, et al. Removing electroencephalographic artifacts by blind source separation. Psychophysiology. 2000;37(2):163-78.

22. Delorme A, Makeig S. EEGLAB: an open source toolbox for analysis of single-trial EEG dynamics including independent component analysis. J Neurosci Methods. 2004;134(1):9-21.
