# Supplementary material for: Mitochondrial, exosomal miR137-COX6A2 and gamma synchrony as biomarkers of parvalbumin interneurons, psychopathology, and neurocognition in schizophrenia
Source: Mol Psychiatry. 2021 Oct 22;27(2):1192–204. doi: 10.1038/s41380-021-01313-9 (PMC9054672; doi:10.1038/s41380-021-01313-9)
Supplement: Supplementary file 2 — Figure and supplementary figure and table Legends [file 41380_2021_1313_MOESM2_ESM.docx]

**Figure Legends**

**Figure 1. Observations in *Gclm-KO* mice:**

**A. Redox dysregulation affects mitochondrial morphology and reduces the expression of mitophagy markers in ACC.** (a) 8-oxo-dG labeling of mitochondrial DNA in the ACC. Scale bar 20μm. (b) Electron micrographs show mitochondrial morphology and mitophagosomes (red arrowheads) in *Gclm*-KO and *Gclm*-WT mice. Scale bar 200nm. (c) Time course of pharmacological challenge with GBR (5mg/kg, s.c. inj.) followed by MitoQ treatment (500µmol/L, drinking water). (d-e) Mitophagy markers (Nix, Fundc1 and LC3B) labeling in ACC across four different groups. Scale bar 50μm. p<0.05*, see representative micrograph in SI (Fig.S1A).

**B. Expression of miR-137 and PV in ACC neurons.** (a) Representative micrographs of miR-137 labeling in ACC across four different groups. Scale bar 50μm. (b) Micrographs showing double labeling for PV (green) and miR-137 (red) in ACC in *Gclm-KO+GBR* and *Gclm-WT+PBS* mice at P40. Scale bar 50μm. (c) Representative micrographs of PV labeling in ACC across the four different groups. Scale bar 50μm. (d) The increased plasmatic levels of miR-137 in mice *Gclm* KO±GBR) was rescued by mitoQ treatment (*Gclm* KO+GBR+MitoQ) back to baseline level, as observed in control animals (*Gclm* WT+PBS). (e) Positive correlation between miR-137 plasma and brain levels in *Gclm* KO+GBR (center panel), reversed by mitoQ treatment (*Gclm* KO+GBR+MitoQ; right panel). p<0.01**, p<0.05*.

**C. Expression of COX6A2 in PV neurons in the ACC.** (a) Colocalization labeling for COX6A2 (red) and PV (green) in ACC was increased in *Gclm* KO+GBR after mitoQ treatment. Scale bar 50 μm. (b) Decreased COX6A2 labeling intensity and COX6A2 / PV colocalization in ACC of P40 mice with or without GBR. (c-d) Local injection of miR-137 inhibitor (i-mmu-miR-137; miRCURY inhibitor Qiagen) in one hemisphere and scramble miR in the contralateral side; immunolabeling for COX6A2 (purple), PV (green), 8-oxo-dG (red) and Dapi (blue) in ACC of *Gclm* KO+GBR mice (c). Inhibition of miR-137 led to decreased oxidative stress marker 8-oxo-DG and increased PVI and COX6A2 staining in *Gclm*-KO+GBR as compared to sham injection (d). p<0.001***, p<0.01**, p<0.05***.**

**Figure 2: Observations in early psychosis patients (EPP)**

**A. Alterations in exosomal markers.** (a) Schema depicting plasma exosomal component. (b) Plasma levels of exosomal miR-137 are elevated in EPP subjects as compared to healthy controls. (c) Mitophagy markers NIX, FUNDC1 and LC3B decreased in EPP subjects. (d) Exosomal plasma COX6A2 levels lower in EPP subjects than in controls. (e) Colocalization (arrows; zoomed panel) of COX6A2 and parvalbumin in exosomes of neuronal origin (L1CAM staining). HSP70 staining: exosomal marker (brain and not brain-derived exosomes). p<0.001***, p<0.01**, p<0.05*

**B. Impaired 40-Hz auditory steady-state response in EPP.** Topographic maps (a) display the averaged evoked power (ePOW) activity between 38-42 Hz and during the entire ASSR period (0-500 ms) for both CTRL and EPP. Responses depict maximal activities measured at (b) the frontocentral ROI (Fz-FCz-Cz) for ePOW (c―e) and intertrial phase coherence (ITC; f―h). Time-frequency maps represent (c) ePOW and (f) ITC at FCz, where color scales indicate ePOW and ITC amplitude values. Line plots show the time course of (d) ePOW and (g) ITC values at the frontocentral ROI (averaged activities from Fz-FCz-Cz) from baseline to post-stimulation for EPP (pink) and CTRL (blue). Boxplots report significant (e) ePOW and (h) ITC differences between CTRL and EPP at the frontocentral ROI for total mean activity (0-500 ms), as well as early- (0-100 ms) and late-latency (300-500 ms) responses (Fig.S5). p<0.01**, p<0.05*, p<0.07^Δ^

**C. Exosomal levels of miR-137 and COX6A2 as surrogate markers of PV alteration.** (a) Exosomal miR-137 levels and late-latency gamma oscillations in the frontocentral ROI recording sites are negatively associated in EPP subjects but not in controls. (b) Exosomal COX6A2 protein levels associated positively with both late-latency ITC and ePOW in healthy controls but not in EPP (values at frontocentral ROI=averaged activities from Fz-FCz-Cz).

**Figure 3: Patient segregation using combined detection of exosomal levels of COX6A2 and miR-137.**

**A.** (a―c) Combined use of miR-137 and COX6A2 exosomal levels allows the classification of “Psy-D” psychosis patients with mitochondrial dysfunction and “Psy-ND” with no/low mitochondrial dysfunction, relative to control subjects. (b) Response probability of the cohort partition using the cutoff points of the miR-137 and COX6A2 expression levels (for more details, see Fig.S6). (c) The plasma levels of exosomal miR-137 and COX6A2 are significantly altered in Psy-D group compared with that in Psy-ND group and in healthy control subjects. (d) Based on the combined detection of miR-137 and COX6A2 levels in blood samples, ROC analysis showed an area under the curve (AUC) of 0.96 for the Psy-D group and a smaller AUC for the Psy-ND and control subjects, showing that the combined detection of miR-137 exosomal level and COX6A2 protein level allows a specific identification of Psy-D patients. (e) Low exosomal COX6A2 protein levels are associated with high miR-137 exosomal concentrations in the Psy-D group.

**B. Impaired ASSR and redox dysregulation in EPP is associated with elevated miR-137 exosomal levels and low exosomal COX6A2 protein levels.** (a) Both ITC and evoked power were decreased in the Psy-D psychosis patients compared with those in the Psy-ND psychosis subjects and control group. Psy-ND and control groups are at the same level. (b) Correlations between GPx and GR activity were only observed in the Psy-ND psychosis subjects and control subjects. (c) No difference was observed in the levels of GPx and GR between groups.

**Figure 4: Cognitive deficits and symptom severity in relation to the stratification based on the combined detection of miR-137 and COX6A2 levels** (A) Boxplots displaying neurocognitive domains (speed processing, attention/vigilance, working memory, problem solving and verbal and visual learning) according to the stratification Psy-D (with mitochondrial dysfunction) and Psy-ND (with no/low mitochondrial dysfunction). Psy-D subjects exhibit worse performance in cognitive tests than Psy-ND subjects and healthy controls. (B) Correlations between neurocognitive and functional profiles and exosomal levels of miR-137 and COX6A2. (C) Psy-D individuals displayed lower GAF, and SOFAS scores than Psy-ND individuals. (D) Psy-D subjects presented more symptoms on the PANSS scale than Psy-ND psychosis subjects. (E) Exosomal levels of miR-137 in the Psy-D individuals (red dots) positively correlated with symptoms severity while Psy-ND (empty circles) do not. (F) Exosomal levels of COX6A2 in the Psy-D individuals negatively correlated with symptoms severity. p<0.001*** p<0.01**, p<0.05*, p<0.07^Δ^

**Figure 5:**  Schematic representation of our reverse translational findings in mice and patients: they reveal that exosomal mitophagy alterations are linked to oxidative stress-induced impairments of parvalbumin interneuron (PVI)-microcircuit in association with gamma oscillation alterations and cognitive deficit. In prefrontal cortex and in blood of redox dysregulated mice (Gclm-KO+GBR), oxidative stress induces miR-137 upregulation, leading to decreased COX6A2 and mitophagy markers, including NIX, Fundc1 and LC3B, and to accumulation of damaged mitochondria. This would further exacerbate oxidative stress and PVI impairments in a positive feedforward process. MitoQ, a mitochondria-targeted antioxidant, rescued all these processes. Translating to early psychosis patients, blood exosomal miR-137 increases and COX6A2 decreases, combined with mitophagy markers alterations suggest that observations made centrally and peripherally in the animal model were reflected in patients’ blood. Higher exosomal miR-137 and lower COX6A2 levels were also associated with a reduction of ASSR gamma oscillations in EEG. As ASSR requires proper PVI-related networks, alterations in combined miR-137/COX6A2 plasma exosome levels may represent a proxy marker of PVI cortical microcircuit impairment that are critically involved in SZ psychopathology and cognition. Blue line: positive correlations; red line: negative correlations; black dashed line: loss/gain association.

**Supplementary Figures**

**Supplementary Figure 1:** A. Representative Micrographs showing mitophagy marker (Nix, Fundc1 and LC3B) labeling in the ACC across the different groups. Scale bar is 50 μm. B. Zoomed high-resolution images of Nix staining.

**Supplementary Figure 2:** Zoomed high-resolution Z-stack images of miR-137 staining.

**Supplementary Figure 3:** miR-137 plasma quantification in the all 8 groups of mice: The effect of mitoQ in plasma of all our 8 experimental groups. The increased plasmatic levels of miR-137 in mice with developmental redox dysregulation (Gclm KO+PBS), which was worsened by an additional oxidative insult (Gclm KO+GBR), was rescued by mitoQ treament (Gclm KO+GBR+mitoQ) back to baseline level, as observed in control animals (Gclm WT+PBS). No difference between the 4 WT groups (PBS/ GBR; +/- MitoQ).

**Supplementary Figure 4:** Expression of COX6A2 in PV neurons in the ACC of *Gclm-KO* mice. Micrographs of double labeling for PV (green) and COX6A2 (red) in the ACC in in the brain of *Gclm*-WT mouse showing colocalization of COX6A2 and parvalbumin immunoreactivities.

**Supplementary Figure 5: Impaired 40-Hz auditory steady-state response in EPP during early and late ASSR responses.** Topographic maps display the averaged evoked power (ePOW) activity between 38-42 Hz and during the early- (0-100 ms) and late-latency (300-500 ms) periods for both CTRL and EPP. Boxplots report significant ePOW and ITC differences between CTRL and EPP at the frontocentral ROI for early- (0-100 ms) and late-latency (300-500 ms) responses. p<0.01**, p<0.05*, p<0.07^Δ^

**Supplementary Figure 6:** Partition for COHORT: Patient-clustering validation.

**Supplementary Figure 7:** CPz and psychosis duration in Psy-D and Psy-ND. No difference was observed in CPZ-equivalent dose of antipsychotics and illness duration between EPP subgroups.

**Supplementary Table 1:** Demographics table. Summary of demographic and clinical characteristics of patients with early psychosis and their matched healthy controls in both the Main cohort (blood samples) and EEG subcohort (EEG – ASSR cohort). Data are reported as the means (±SDs) or percentages. Results of T-tests and Chi-squared tests (test values, df and p-values) are reported when differences between patients and controls were tested. Note: CPZ eq., chlorpromazine equivalent (antipsychotic medication, Fig.S7); PANSS, Positive and Negative Syndrome Scale; GAF, Global Assessment of Functioning; EEG-ASSR-40, electroencephalographic auditory steady-state response at 40 Hz.

**Supplementary Table 2:** List of markers used for immunohistochemistry or Western blot and characteristics of their corresponding antibodies.

**Supplementary Table 3:** Demographic and clinical characteristics of early psychosis patients with (Psy-D) or without mitochondrial dysregulation (Psy-ND). ^a:^Number of cigarettes smoked per day; ^b:^ Case Manager Rating Scale.

**Supplementary Table 4:** Patient antipsychotic medications. Antipsychotics from the main cohort with blood samples and the sub-cohort with EEG-ASSR data. Data are reported as percentages (numbers of patients).

**Supplementary table 5:** Detailed medication list after stratification of EPP main cohort. There is no difference in the percentage of patients taking antipsychotics and other medication between the PsyD and Psy-ND groups. The percentage of patients taking other non-antipsychotic medication is very small, except for the anxiolytic “Lorazepam” (ca. 30% in both groups). Data are reported in terms of percentage of total of patients of each group (n). The total percentage in each column exceeds 100%, as many patients have multiple medication. *PRN: pro re nata.

**Supplementary table 6:** Comparison of correlations using Fisher's r to z transformation. Z scores were compared and analyzed for statistical significance (p value).
