# Supplementary figures and images for: Mitochondrial, exosomal miR137-COX6A2 and gamma synchrony as biomarkers of parvalbumin interneurons, psychopathology, and neurocognition in schizophrenia
Source: Mol Psychiatry. 2021 Oct 22;27(2):1192–204. doi: 10.1038/s41380-021-01313-9 (PMC9054672; doi:10.1038/s41380-021-01313-9)

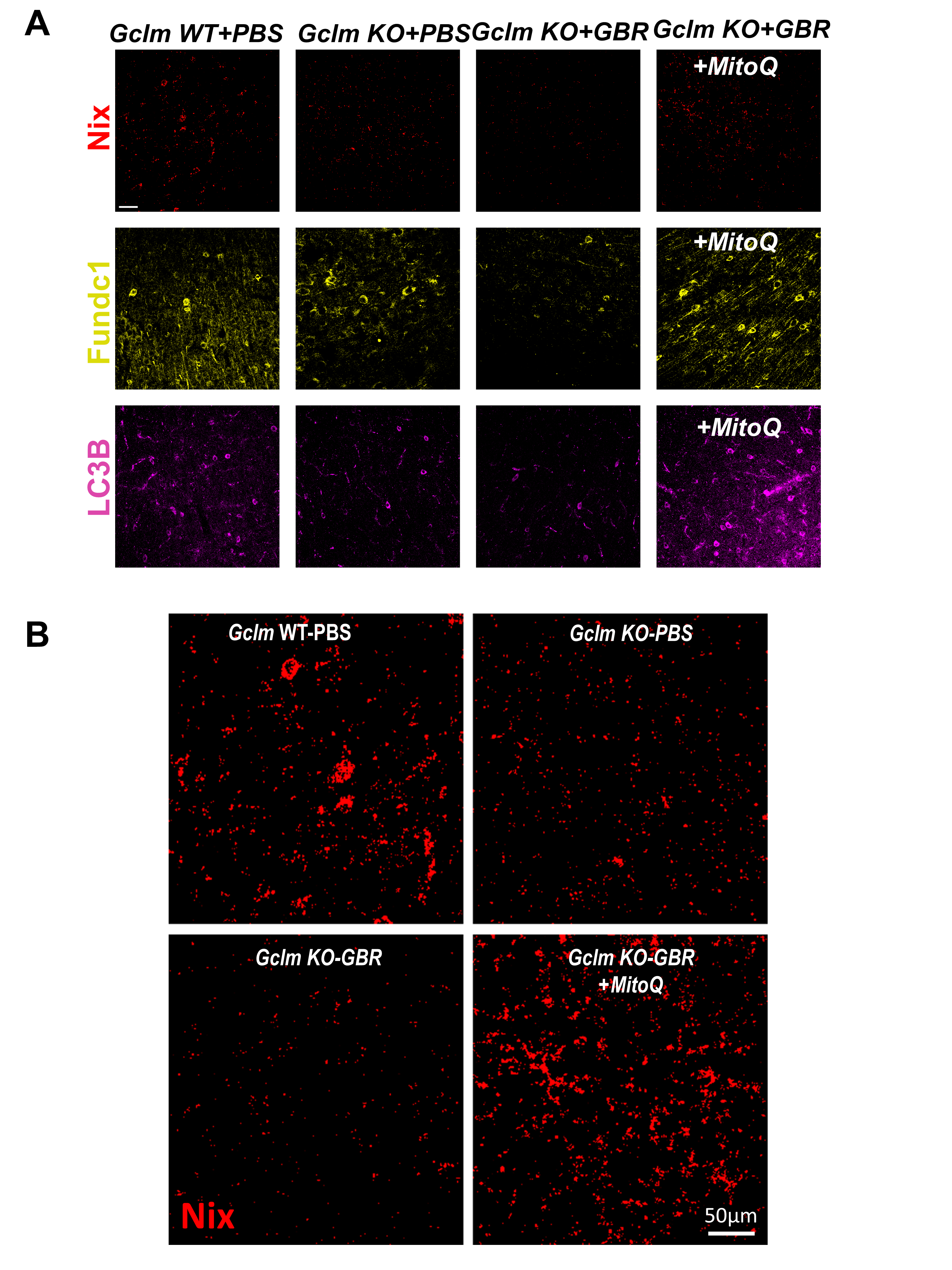

Supplement: Supplementary file 3 — Supplementary Figure 1 [file 41380_2021_1313_MOESM3_ESM.tif]

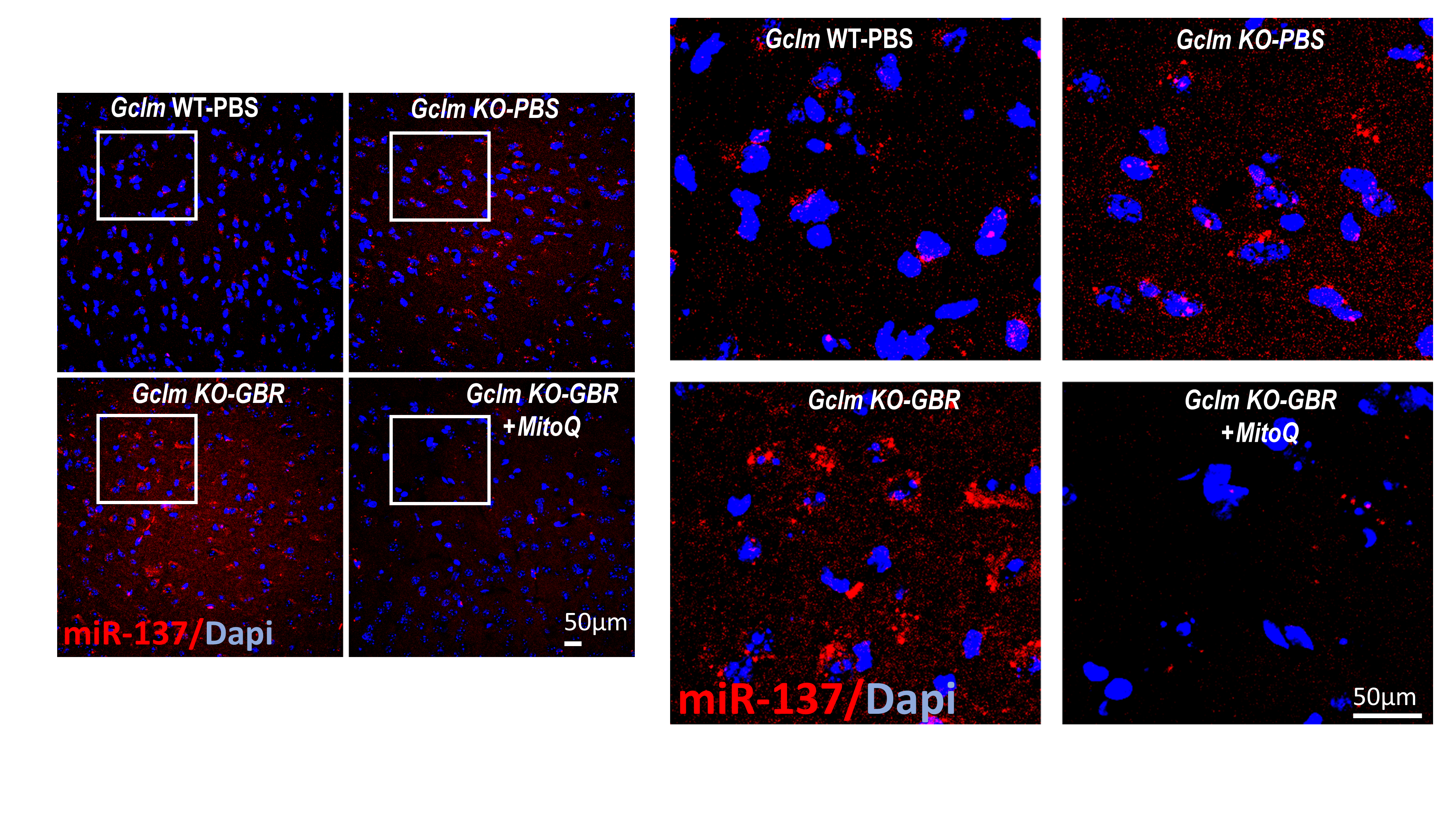

Supplement: Supplementary file 4 — Supplementary Figure 2 [file 41380_2021_1313_MOESM4_ESM.tif]

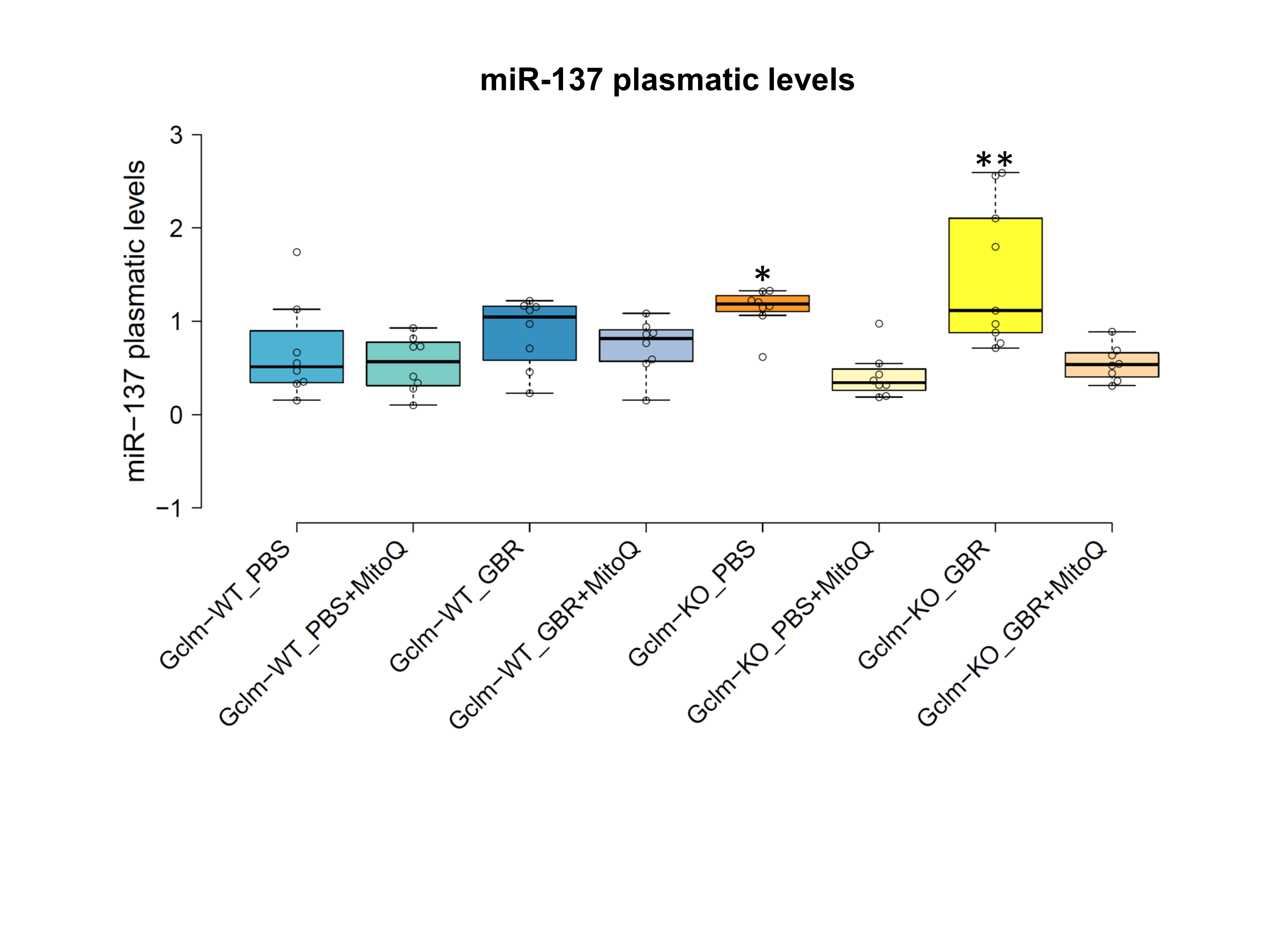

Supplement: Supplementary file 5 — Supplementary Figure 3 [file 41380_2021_1313_MOESM5_ESM.tif]

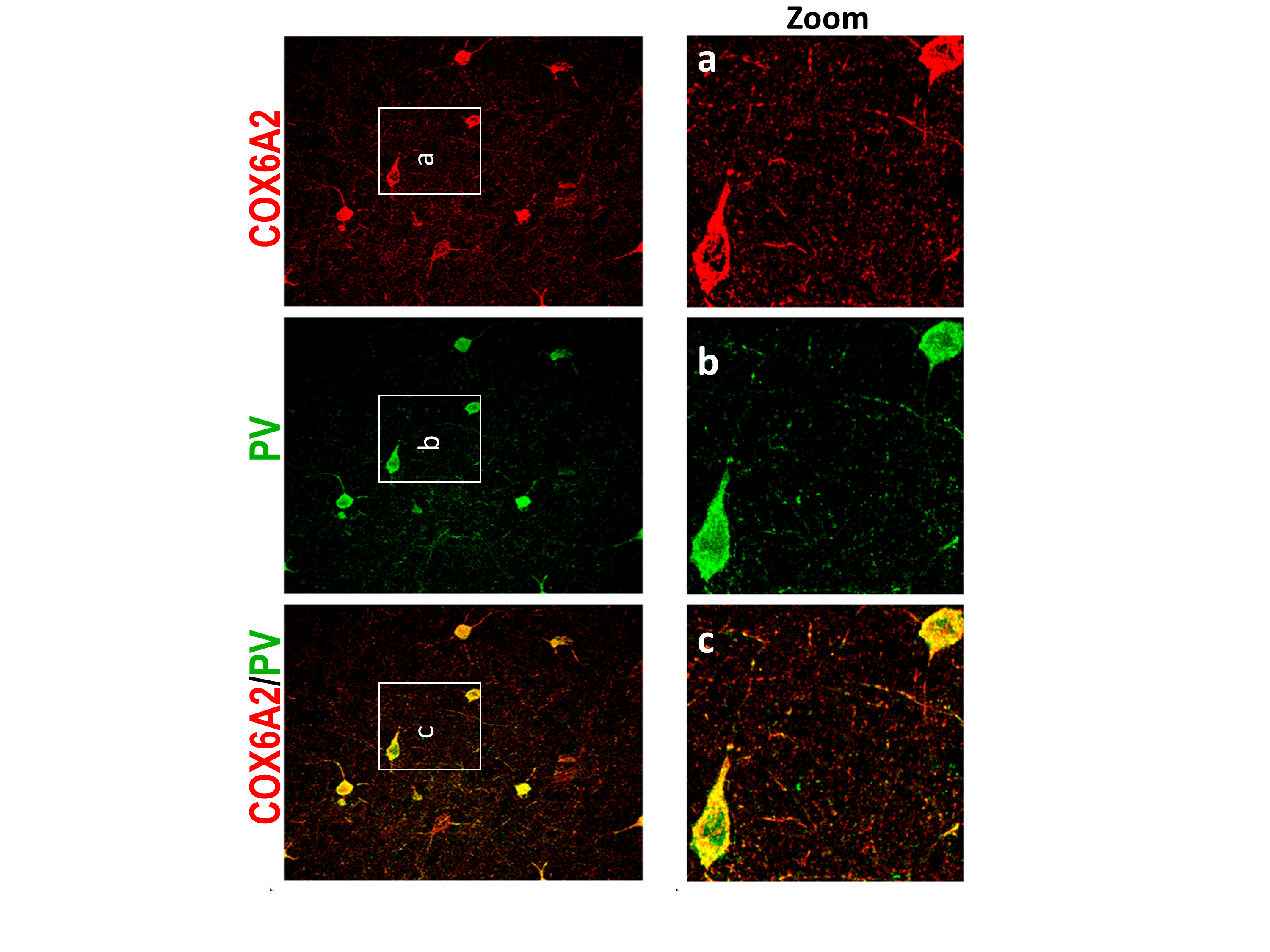

Supplement: Supplementary file 6 — Supplementary Figure 4 [file 41380_2021_1313_MOESM6_ESM.tif]

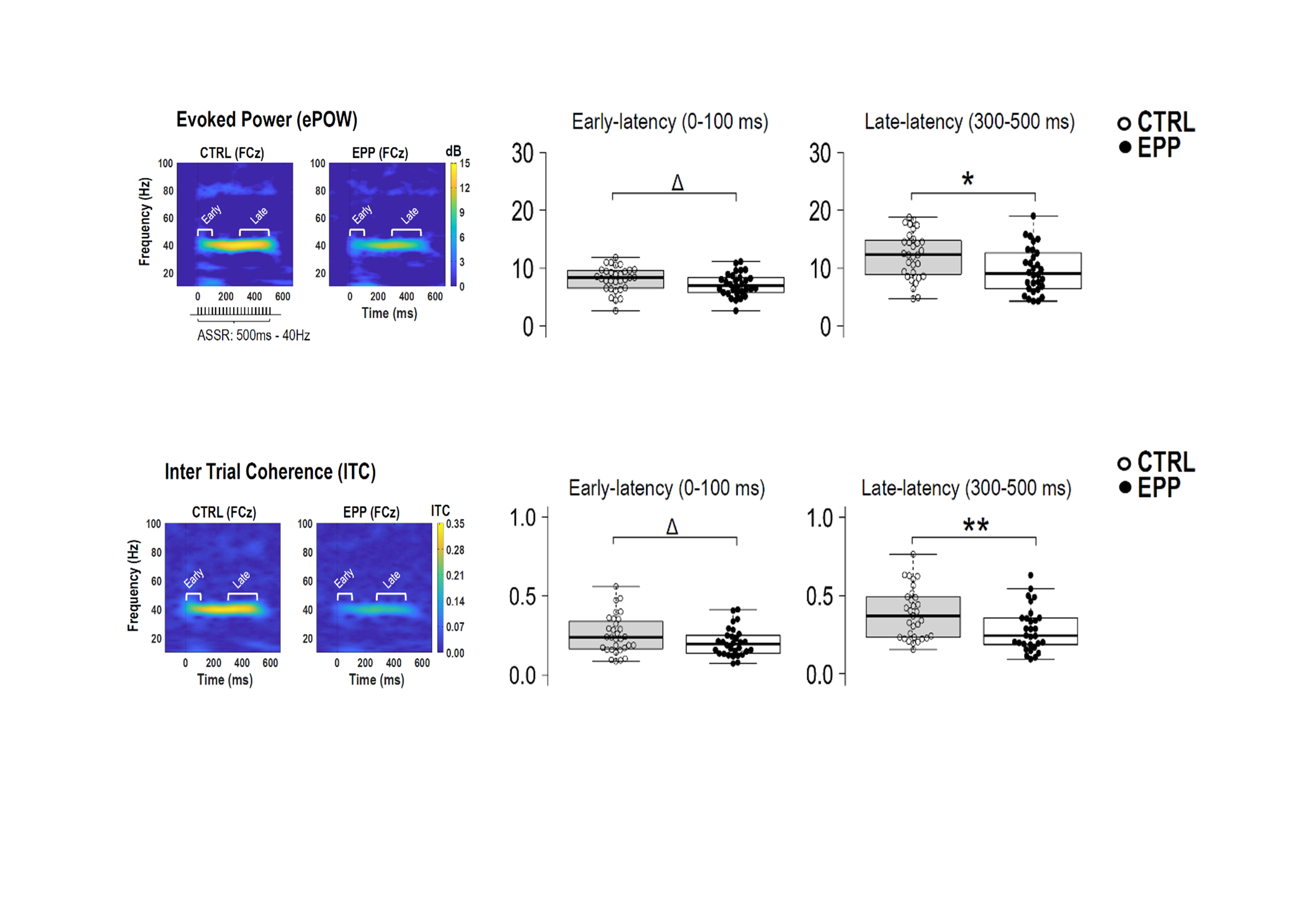

Supplement: Supplementary file 7 — Supplementary Figure 5 [file 41380_2021_1313_MOESM7_ESM.tif]

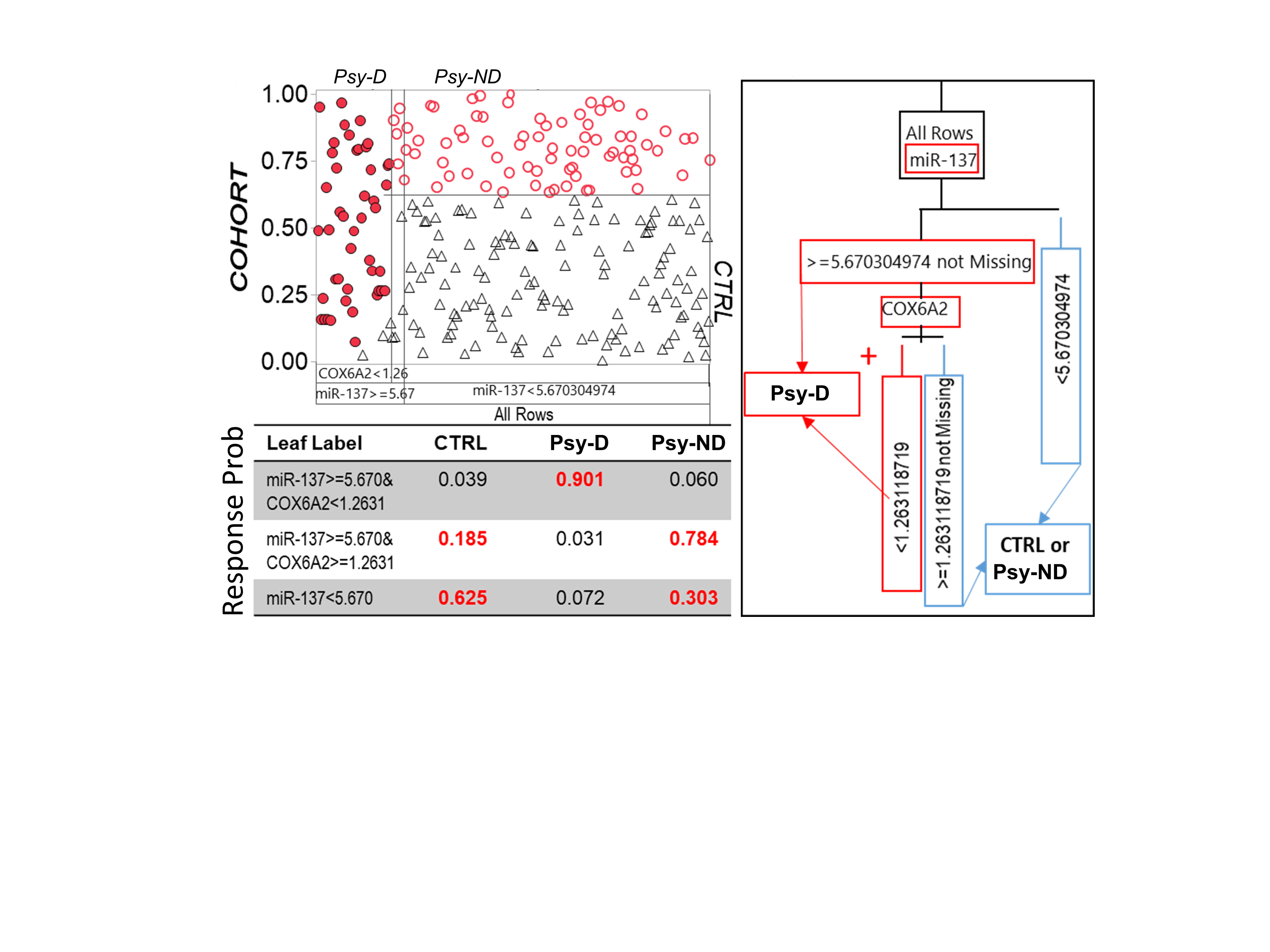

Supplement: Supplementary file 8 — Supplementary Figure 6 [file 41380_2021_1313_MOESM8_ESM.tif]

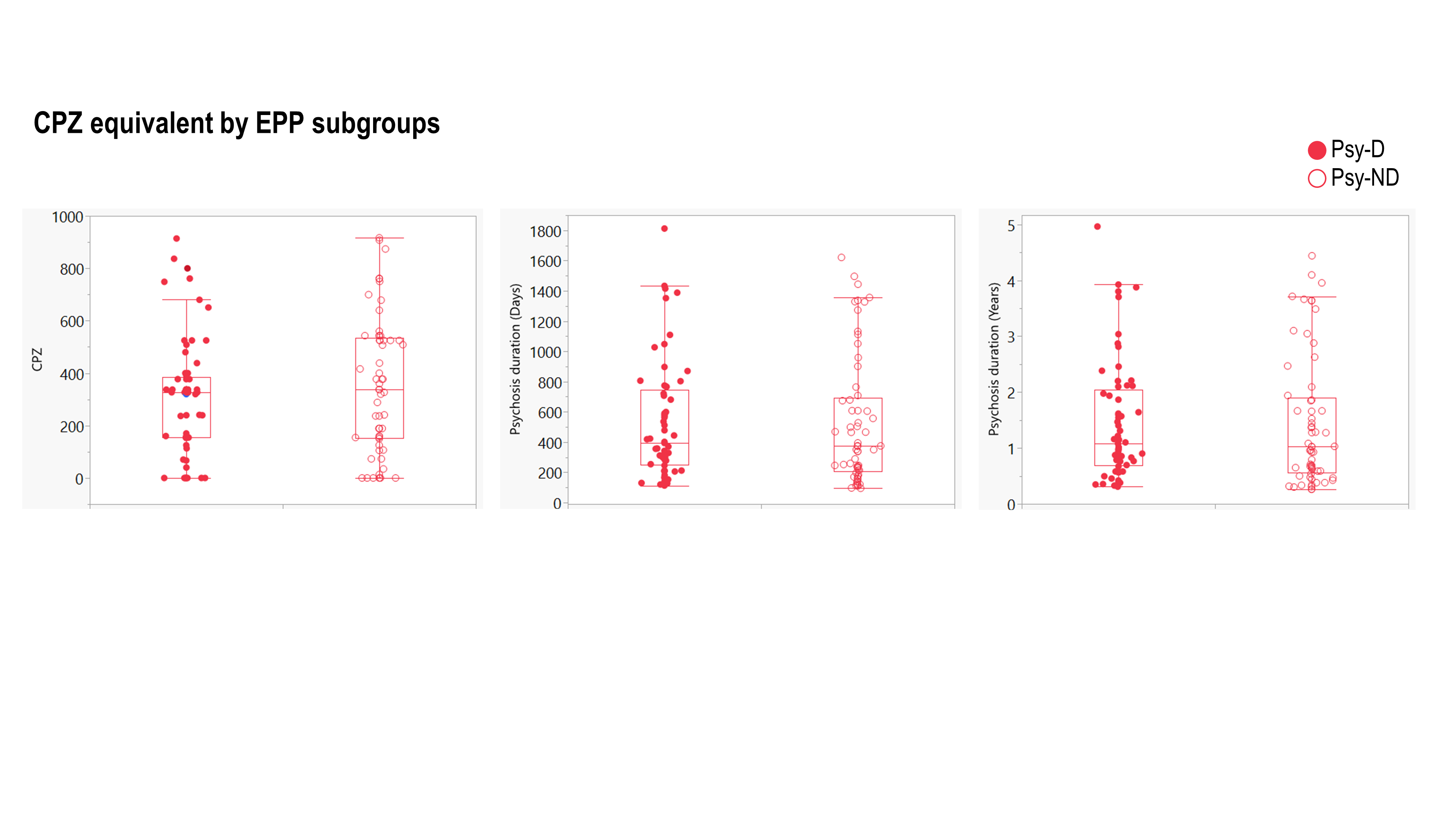

Supplement: Supplementary file 9 — Supplementary Figure 7 [file 41380_2021_1313_MOESM9_ESM.tif]

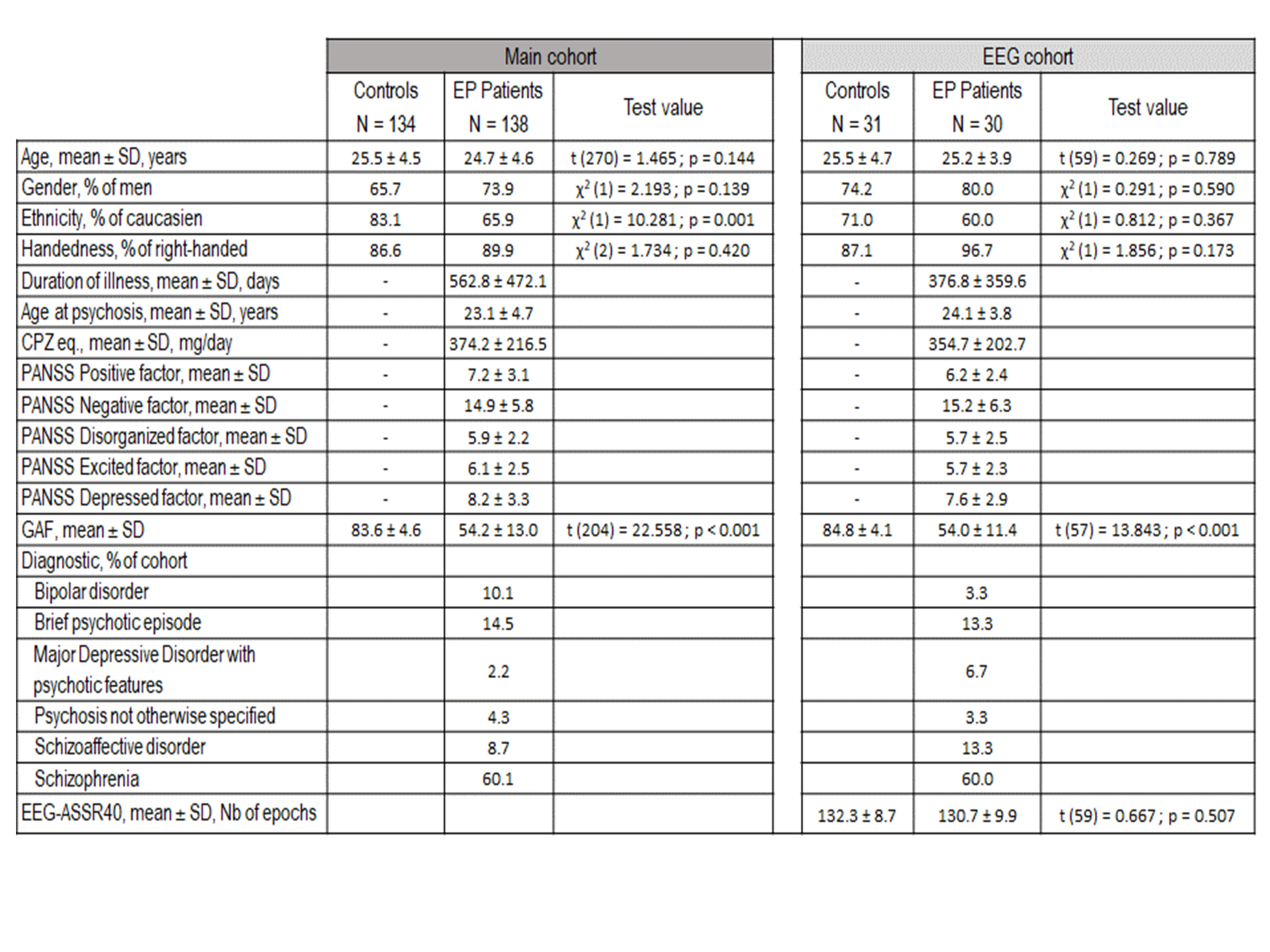

Supplement: Supplementary file 10 — Supplementary Table 1 [file 41380_2021_1313_MOESM10_ESM.tif]

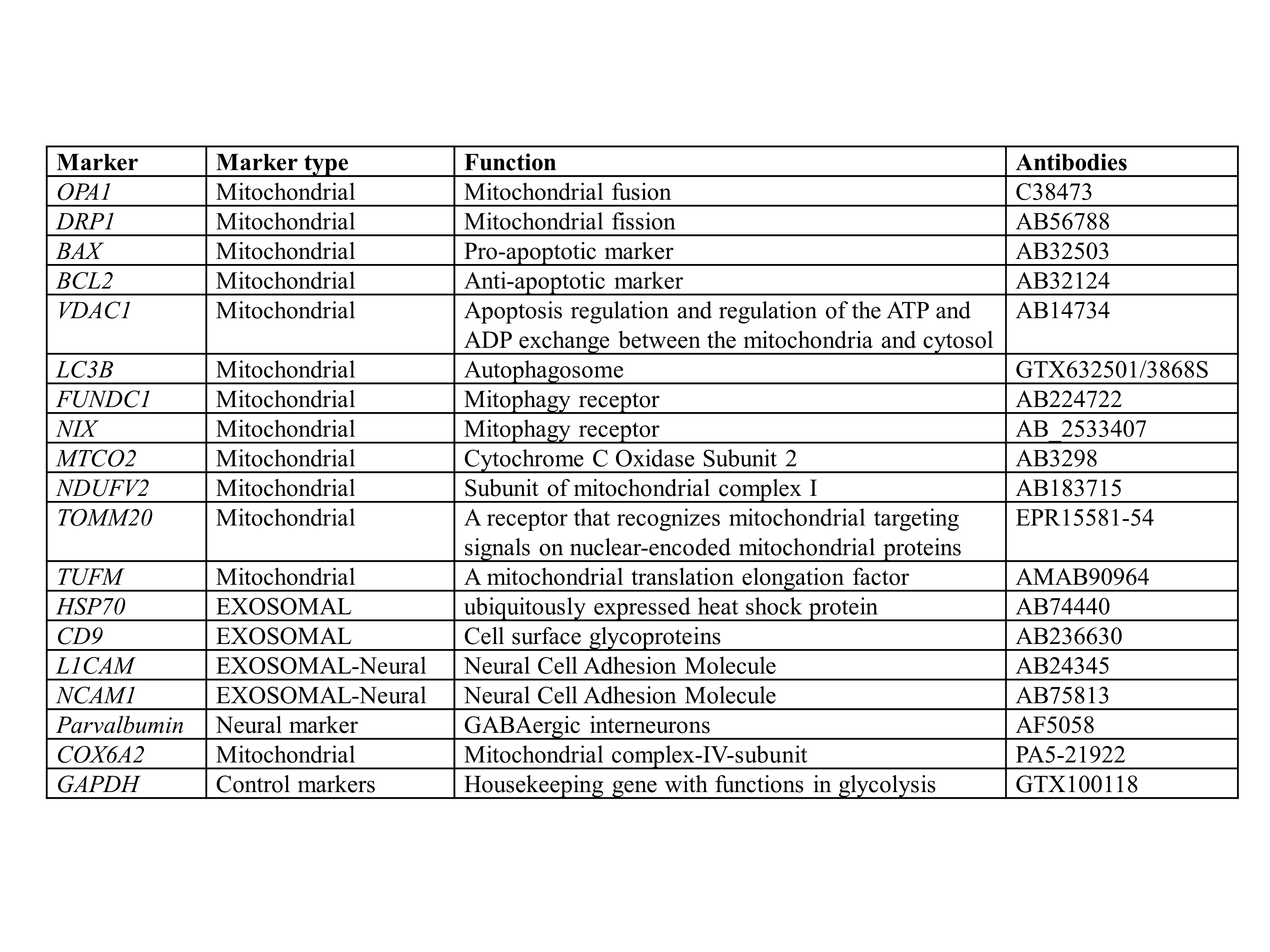

Supplement: Supplementary file 11 — Supplementary Table 2 [file 41380_2021_1313_MOESM11_ESM.tif]

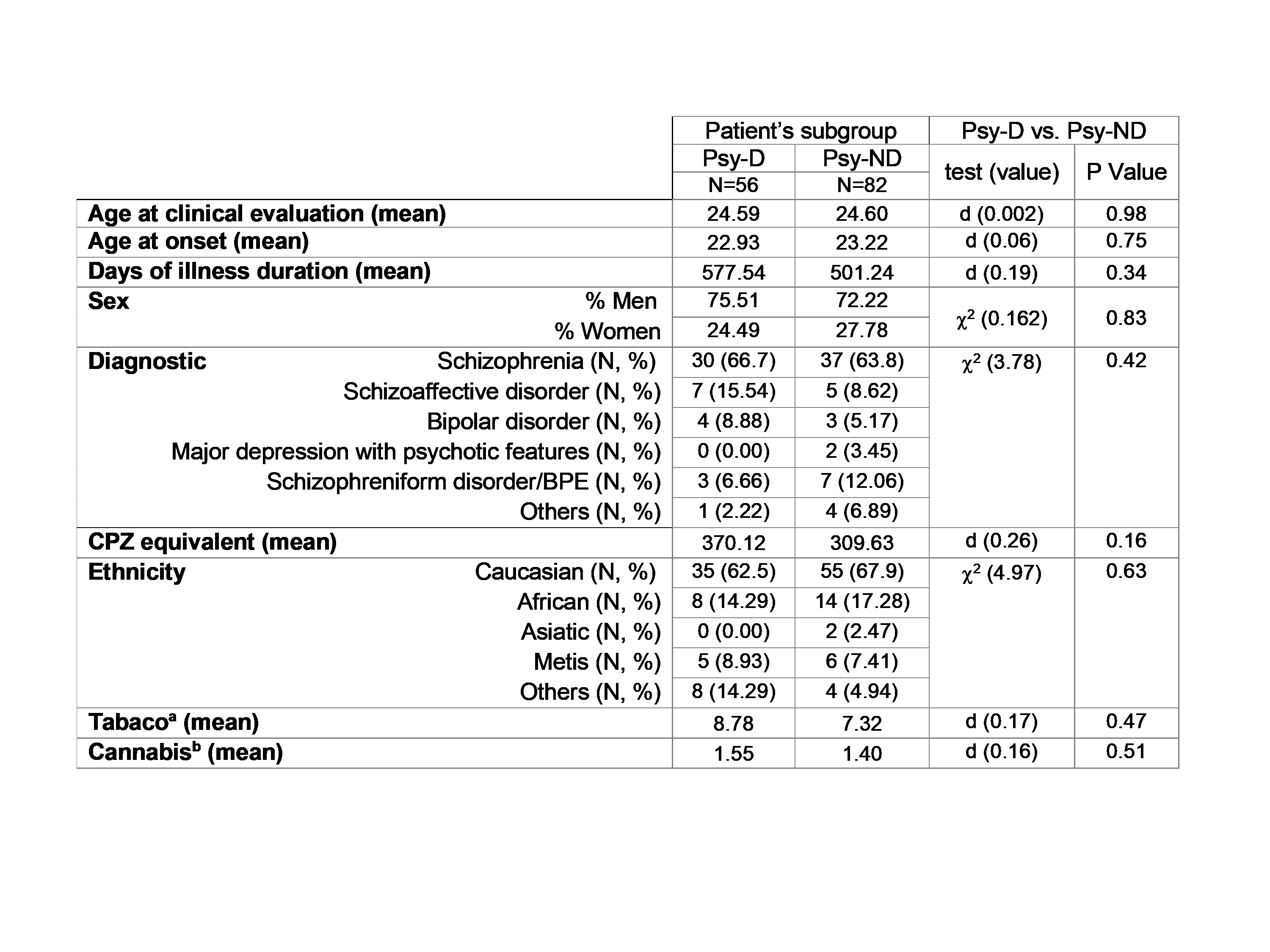

Supplement: Supplementary file 12 — Supplementary Table 3 [file 41380_2021_1313_MOESM12_ESM.tif]

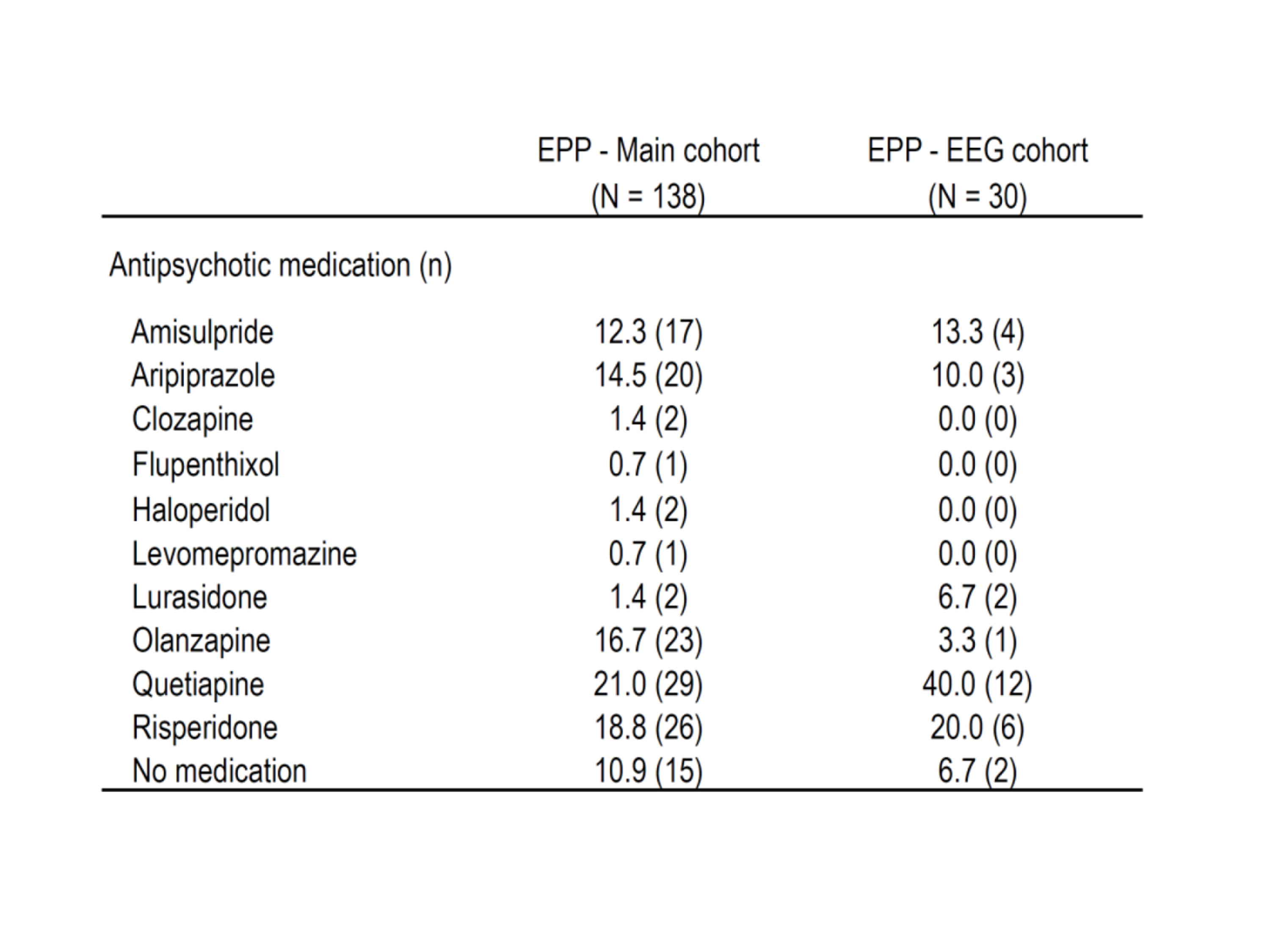

Supplement: Supplementary file 13 — Supplementary Table 4 [file 41380_2021_1313_MOESM13_ESM.tif]

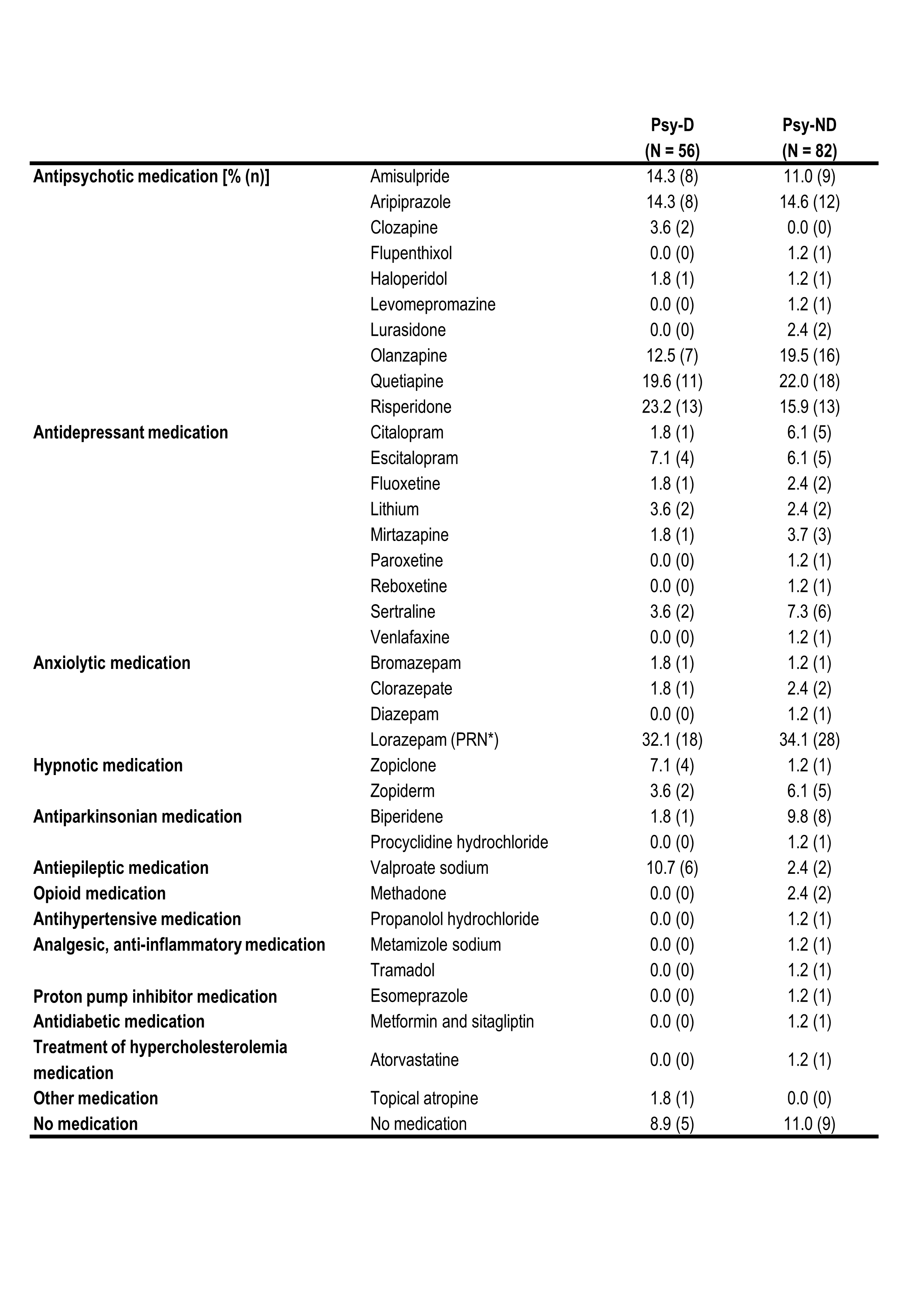

Supplement: Supplementary file 14 — Supplementary Table 5 [file 41380_2021_1313_MOESM14_ESM.tif]

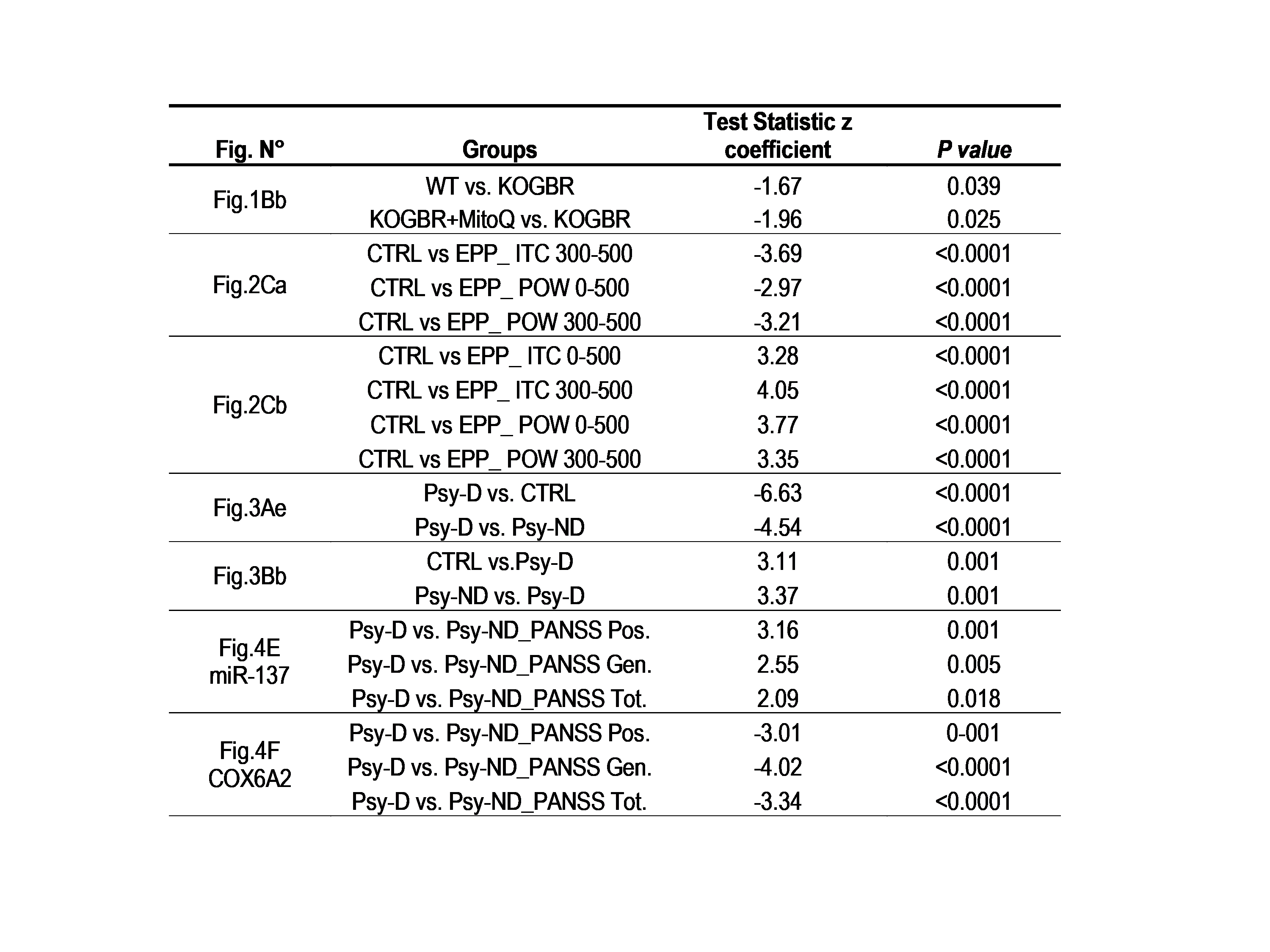

Supplement: Supplementary file 15 — Supplementary Table 6 [file 41380_2021_1313_MOESM15_ESM.tif]
